# Improved *de novo* genome assembly and analysis of the Chinese cucurbit *Siraitia grosvenorii*, also known as monk fruit or luo-han-guo

**DOI:** 10.1093/gigascience/giy067

**Published:** 2018-06-08

**Authors:** Mian Xia, Xue Han, Hang He, Renbo Yu, Gang Zhen, Xiping Jia, Beijiu Cheng, Xing Wang Deng

**Affiliations:** 1 Key Laboratory of Crop biology of Anhui Province, Anhui Agricultural University, Hefei, China; 2 School of Advanced Agriculture Sciences and School of Life Sciences, State Key Laboratory of Protein and Plant Gene Research, Peking University, Beijing 100871, China; 3 National Demonstration Area of Modern Agriculture in Cangxi, Sichuan Province, China

**Keywords:** *Siraitia grosvenorii*, monk fruit, PacBio sequencing, ortholog analysis, RNA-Seq, mogrosides biosynthesis

## Abstract

**Background:**

Luo-han-guo (*Siraitia grosvenorii*), also called monk fruit, is a member of the Cucurbitaceae family. Monk fruit has become an important area for research because of the pharmacological and economic potential of its noncaloric, extremely sweet components (mogrosides). It is also commonly used in traditional Chinese medicine for the treatment of lung congestion, sore throat, and constipation. Recently, a single reference genome became available for monk fruit, assembled from 36.9x genome coverage reads via Illumina sequencing platforms. This genome assembly has a relatively short (34.2 kb) contig N50 length and lacks integrated annotations. These drawbacks make it difficult to use as a reference in assembling transcriptomes and discovering novel functional genes.

**Findings:**

Here, we offer a new high-quality draft of the *S. grosvenorii* genome assembled using 31 Gb (∼73.8x) long single molecule real time sequencing reads and polished with ∼50 Gb Illumina paired-end reads. The final genome assembly is approximately 469.5 Mb, with a contig N50 length of 432,384 bp, representing a 12.6-fold improvement. We further annotated 237.3 Mb of repetitive sequence and 30,565 consensus protein coding genes with combined evidence. Phylogenetic analysis showed that *S. grosvenorii* diverged from members of the Cucurbitaceae family approximately 40.9 million years ago. With comprehensive transcriptomic analysis and differential expression testing, we identified 4,606 up-regulated genes in the early fruit compared to the leaf, a number of which were linked to metabolic pathways regulating fruit development and ripening.

**Conclusions:**

The availability of this new monk fruit genome assembly, as well as the annotations, will facilitate the discovery of new functional genes and the genetic improvement of monk fruit.

## Data Description

### Introduction

**Figure 1:**
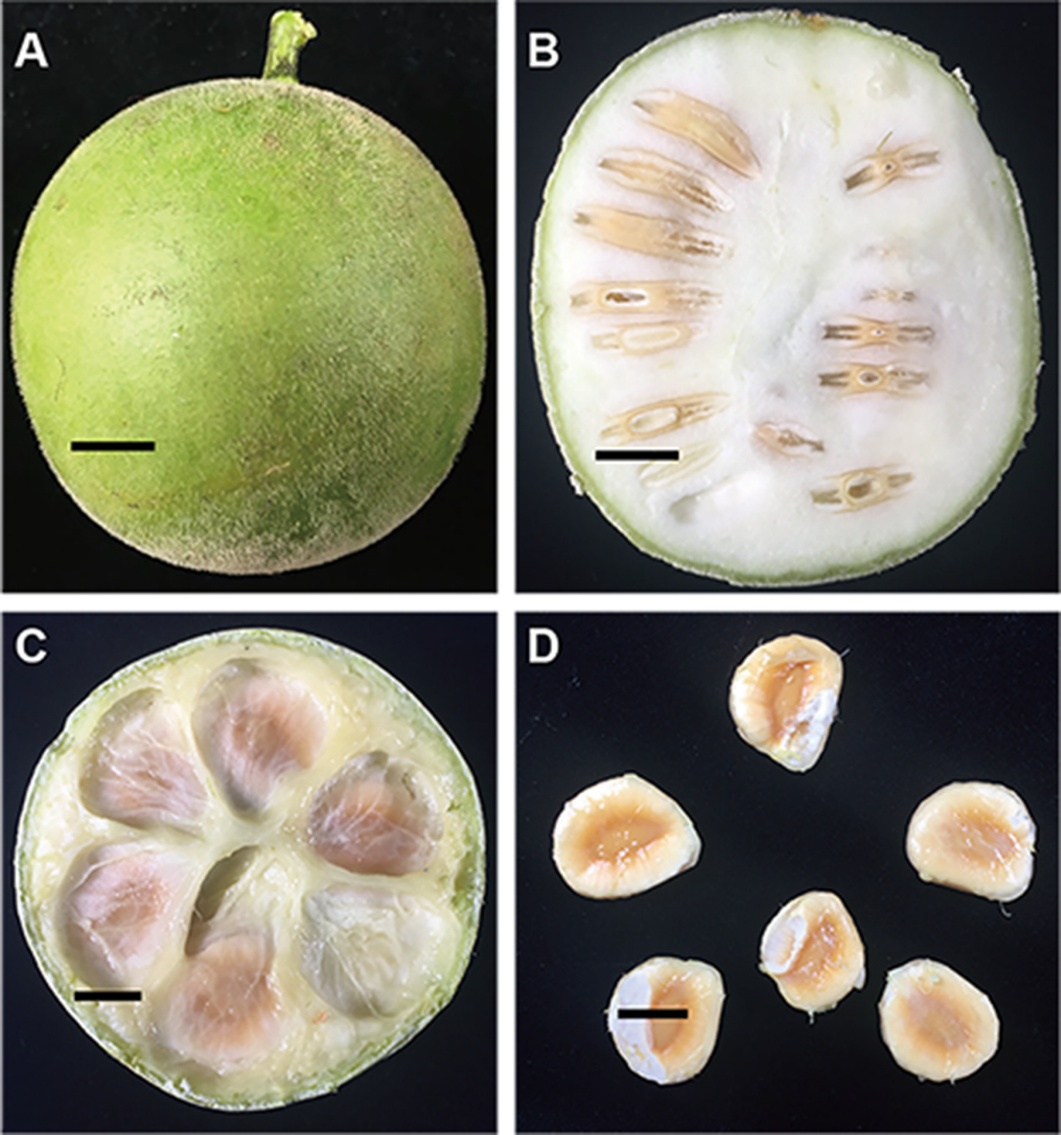
Morphological characteristics of the fruit of *S. grosvenorii (A)*, vertical section of fruit of *S. grosvenorii* (B), horizontal section of fruit of *S. grosvenorii* (C) and seeds (D). Size bar, 1 cm.

*Siraitia grosvenorii* (luo-han-guo or monk fruit, NCBI Taxonomy ID: 190515) is an herbaceous perennial native to southern China and is a famous specialty in Guilin city, Guangxi Province of China (Fig. 1) [1]. In addition to being used as a natural sweetener, *S. grosvenorii* has been used in China as a folk remedy for the treatment of lung congestion, sore throat and constipation for hundreds of years [2]. The ripe fruit of *S. grosvenorii* contains mogrosides, which have become a popular research topic due to their pharmacological characteristics, including putative anti-cancer properties [3]. Additionally, mogrosides are purified and used as a non-caloric, non-sugar sweetener in the United States and Japan, as they are estimated to be approximately 300 times as sweet as sucrose [1, 4]. To date, *S. grosvenorii* fruit was shown to have additional pharmacological effects and contain different types of secondary metabolites [5, 6]. Monk fruit products have been approved as dietary supplements in Japan, the US, New Zealand and Australia [2, 7].

**Figure 2:**
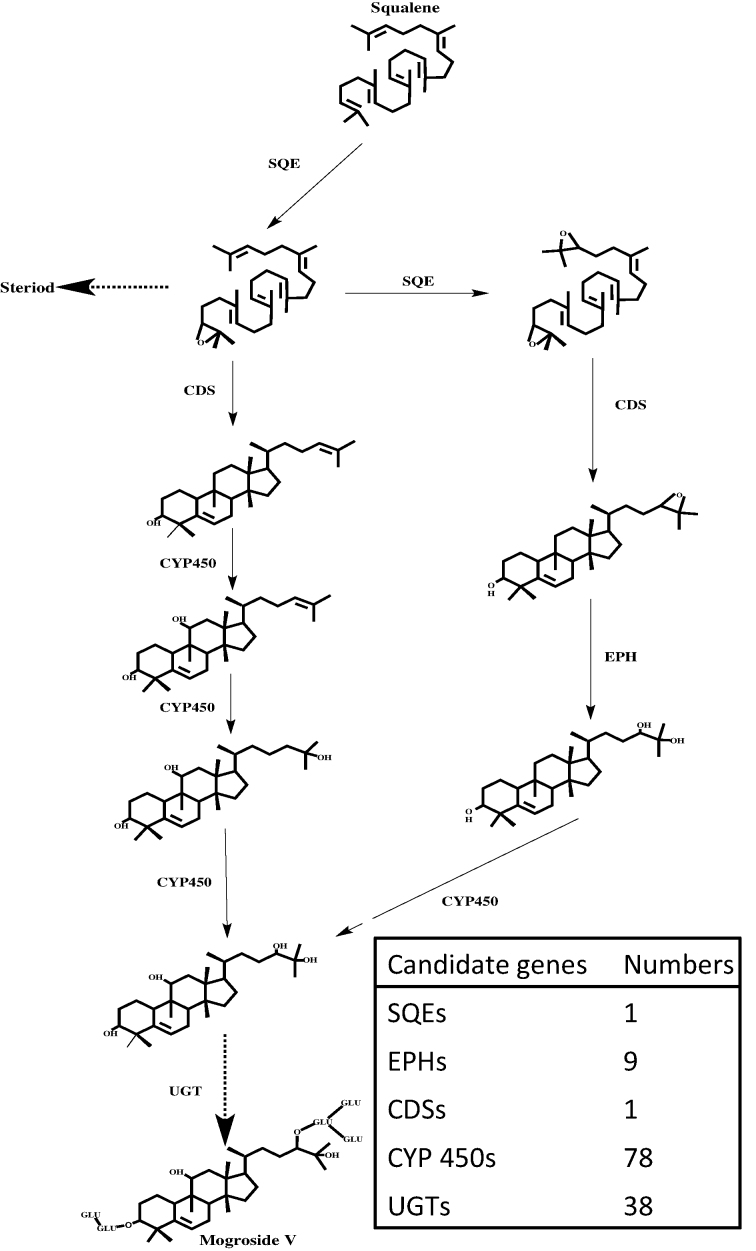
Candidate genes involved in the mogrosides biosynthesis pathway. Candidate functional genes were annotated as SQEs, EPHs, CDSs, CYP450s, and UGTs and assigned to the pathway.

The biosynthesis pathway of mogrosides has been extensively studied, and several genes have been identified [8-11]. Squalene is thought to be the initial substrate and precursor for triterpenoid and sterol biosynthesis. Squalene epoxidases (SQE) perform epoxidation, which creates squalene or oxidosqualene, and cucurbitadinenol synthase (CDS) cyclizes oxidosqualene to form the cucurbitadienol triterpenoid skeleton, which is a distinct step in phytosterol biosynthesis [12]. Epoxide hydrolases (EPH) and cytochrome P450s (CYP450) further oxidize cucurbitadienols to produce mogrol, which is glycosylated by UDP-glycosyl-transferases (UGT) to form mogroside V (Fig. 2).

The genome of *S. grosvenorii* was first published in 2016 and served the purpose of identifying the genomic organization of the gene families of interest but did not act as the reference in the transcriptome assembly and gene families identification [8]. Although the first draft genome assembly was a useful resource, some improvements remain necessary, including improving the continuity and completeness, genome assembly assessment, annotation of genes and repetitive regions, and analysis of other genomic features. With an average read length now exceeding 10 kb, SMRT sequencing technology from Pacific Biosciences (PacBio) has the potential to significantly improve genome assembly quality [13]. Therefore, we *de novo* assembled a high-quality genome draft of *S. grosvenorii* using high-coverage PacBio long reads and applied extensive genomic and transcriptomic analyses. This new assembly, annotations, and other genomic features discussed below will serve as valuable resources for investigating the economic and pharmacological characteristics of monk fruit and will also assist in the molecular breeding of monk fruit.

### DNA libraries construction and sequencing

A total of 20 μg of genomic DNA was extracted from seedlings of *S. grosvenorii* (variety Qingpiguo) using a modified CTAB method [14] to construct two libraries with an insert size of 20 kb. The plants were introduced from the Yongfu District (Guangxi Province, China) and planted in Cangxi County (Sichuan Province, China). Sequencing of *S. grosvenorii* was performed using the Pacbio RSII platform (Pacific Biosciences, USA) and generated 31 Gb (∼73.8 x) of data from 44 SMRT cells, with an average subread length of 7.7 kb and read quality of 82% after filtering out low-quality bases and adapters (Table 1).

**Table 1:** SMRT reads used for genome assembly

Statistics	Length (bp)
Total raw data	31 G
Mean length of raw reads	11 k
N50 of raw reads	15 754
Mean length of subreads	7.7 k
N50 of subreads	11,898

Subreads: reads without adapters and low-quality bases.

A total of 300 ng of genomic DNA was extracted as described above, and the library was constructed using DNA sequence fragments of ∼470 bp, with an approximate insert size of 350 bp. Sequencing was performed using a 2 × 150 paired-end (PE) configuration, and base calling was conducted using the HiSeq Control Software + Off-Line Base Caller (OLB) + GAPipeline-1.6 (Illumina; CA, USA) on the HiSeq instrument, which generated a total of 169 M (over 100 x) short reads.

### RNA isolation and sequencing

Fresh roots, leaves, and early fruit of *S. grosvenorii* were sampled in our garden in Cangxi County. All samples were stored at -80°C after immediate treatment with liquid nitrogen. Total RNA was isolated from (1) leaves of female plants (FL), (2) leaves of male plants (ML), (3) leaves beside fruits (L), (4) roots (R), (5) fruit of 3 DAA (F1), and (6) fruit of 20 DAA (F2) using the Qiagen RNeasy Plant Mini Kits (Qiagen, CA, USA). PE libraries (PE150 with an insert size of 350 bp) were constructed and subsequently sequenced via the Illumina HiSeq X-Ten platform (Illumina, CA, USA).

### Genome assembly

Initial correction of long reads was performed using FALCON (Falcon, RRID:SCR_016089) [15] with _cutoff length = 5,000 according to the distribution of read lengths and -B15, -s400 to cut reads into blocks of 400 Mb and align 15 blocks to another block at the same time. The 25x coverage of the longest corrected reads was extracted with Perl scripts and assembled by mecat2canu command of MECAT [15] with GenomeSize = 420 000 000 estimated in the previous study [8]. This led to a new genome assembly of 467 Mb with a contig N50 size of 434,684 bp (Table 2). This genome size was slightly larger than the estimated 420 Mb [8], which was likely due to the high genome heterozygosity. We used the consensus algorithm Quiver [16] and further polished the assembly with PE reads using Pilon (Pilon, RRID:SCR_014731) [17]. The final assembly produced 4,128 contigs, 614 of which were >100 kb long, with a contig N50 length of 432,384 bp (Table 2). Compared to the preliminary draft of the published *Siraitia* genome, the contiguity was improved more than ∼12.6 times.

**Table 2:** Metrics of *de novo S. grosvenorii* genome assembly

Statistics	Contig	Contig (polished)
Total number	4128	4128
Total length (bp)	467,072,951	469,518,713
N50 length (bp)	433,684	432,384
N90 length (bp)	36,820	36,953
Max length (bp)	7657,852	7683,850
GC content (%)	33.57	33.49

### Genome assessment

We estimated the completeness of the assembly using Benchmarking Universal Single-Copy Orthologues (BUSCO v2, RRID:SCR_015008) [18] analysis. Of the 1,440 orthologues identified in plants, 1,284 were found in the genome assembly, including 849 in single-copy and 435 in multi-copy (Table 3). In addition, we used RNA-Seq data from different organs to assess the sequence quality. All 15 RNA-Seq libraries were mapped to the assembly using HISAT2 (HISAT2, RRID:SCR_015530) [19], and the overall alignment rate for each data was used as a rough estimation of sequence quality. We also estimated the base error rate of the assembly with both DNA paired-end reads and published DNA short reads [8]. We used BWA-mem [20] to align both short reads to the genome assembly and filtered out low-quality (mapping quality <30) alignments with SAMtools (SAMtools, RRID:SCR_002105) [21]. Then, we used the Genome Analysis Toolkit (RRID:SCR_001876) HaplotypeCaller [22] to call short variants. The Genome Analysis Toolkit VariantFiltration program was used to filter out low-quality variants with the following expression: QD < 2.0 || ReadPosRankSum < -8.0 || FS > 60.0 || QUAL < 50 || DP < 10. Coverage of each alignment file was scanned using Qualimap 2 [23], and the error rate was calculated as the average number of short variants that appear at both alleles (labeled as 1/1 and 1/2 in Table 5) per base. The overall alignment rates of reads in all samples were over 80% (Table 4), and the average base error rate was estimated as less than 1E-3, which suggests a high-quality assembly (Table 5).

**Table 3:** Summarized benchmarks of the BUSCO assessment

	Monk fruit (%)
Complete BUSCOs	89.2
Complete and single-copy	59.0
Complete and duplicated	30.2
Partial	2.7
Missing	8.1

**Table 4:** Quality evaluation of the draft genome with the overall alignment rate

Sample	Overall alignment rate
FL-1	89.93%
FL-2	87.75%
FL-3	85.83%
ML-1	89.70%
ML-2	89.73%
ML-3	85.07%
L-1	85.95%
L-2	87.39%
R-1	81.50%
R-2	84.36%
R-3	84.57%
F1–1	84.35%
F1–2	91.58%
F2–1	86.83%
F2–2	87.37%

FL: female leaf, ML: male leaf, L: leaf, R: root, F1: fruit stage 1, F2: fruit stage 2.

**Table 5:** Genome base accuracy estimated using resequencing short reads

			Number of variation				
Sample	Mean depth	Coverage	0/1	1/1	1/2	Total	Error rate
Paired-end	65.3 x	92.99%	1342,849	37,987	14,704	1395,540	1.21E-4
Published	80.0 x	90.79%	2569,592	172,906	16,777	2759,276	4.45E-4

High-quality genome criteria: 1E-4.

0: genotype that is identical to the reference, 1,2: genotype that is different from the reference.

Error rate = (Number of 1/1 + Number of 1/2)/(Genome size * Coverage).

### Repeat annotation

We scanned the genome using RepeatMasker (RRID:SCR_012954) [24] with Repbase [25] and a *de novo* repeat database constructed with RepeatModeler (RID:SCR_015027) [26]. Sequences 240 Mb (51.14% of the assembled genome) in length were identified as repetitive elements, which was slightly larger than the 42.8% of *Momordica charantia* [27] and much larger than the 28.2% of *Cucumis sativus* [28]. We further classified the repetitive regions and found that the vast majority were interspersed repeats. Among them, the main subtypes were unclassified repeats and long terminal repeats (LTRs), with Copia (27.1 Mb, 5.8% of the genome) and Gypsy (38.6 Mb, 8.2% of the genome) LTRs being the most abundant. Compared to cucumber, the genome enlargement in monk fruit and bitter gourd was likely driven by the expansion of interspersed repeats (Table 6).

**Table 6:** Repeat annotation of the *S. grosvenorii* genome

	*S. grosvenorii*		*M. charantia*		*C. sativus*		
Repeat classification		Length (bp)	Content	Length (bp)	Content	Length (bp)	Content
Interspersed repeats	SINEs	0	0.00%	0	0.00%	0	0.00%
	LINEs	9629,949	2.05%	5183,926	1.82%	2397,830	1.22%
	LTR	67,499,840	14.38%	34,217,647	11.98%	8253,090	4.18%
	DNA elements	9372,444	2.00%	3460,431	1.21%	2777,943	1.41%
	Unclassified	147,311,542	31.38%	75,056,338	26.28%	37,539,553	19.03%
	Total	233,813,775	49.80%	117,918,342	41.29%	50,967,966	25.84%
Simple repeats		5401,880	1.15%	3451,508	1.21%	3547,474	1.80%
Low complexity		1570,875	0.33%	958,289	0.34%	1095,406	0.56%
Total		240,122,745	51.14%	122,111,538	42.75%	55,540,243	28.15%

### Gene annotation

**Figure 3:**
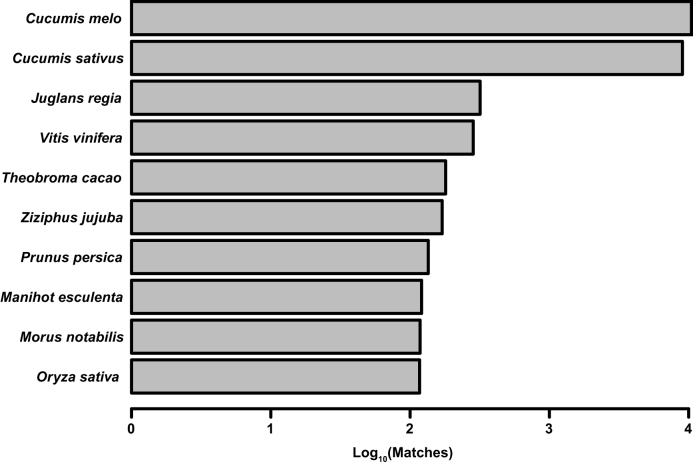
Number of best-matching proteins for each predicted *S. grosvenorii* gene by species.

To generate gene models, the *S. grosvenorii* genome was annotated using three gene prediction pipelines including homology-based, *de novo*, and RNA-Seq data-based prediction. First, we aligned the three cucurbitaceous proteomes downloaded from the cucurbit database ([29] cucumber_Chinese_Long_v2, melon_v3, and watermelon_97 103_v1) to the genome assembly using TBLASTN with an E-value of 1e-5 and filtering out bad hits (identity <50% and length <50%). The best hit of each retained protein was extracted and further used to predict protein coding gene structures with GeneWise (RRID:SCR_015054) [30, 31]. Second, we *de novo* predicted protein coding genes using AUGUSTUS (RRID:SCR_008417) [32] with the repeat masked genome. Third, we used StringTie [33] to assemble 15 RNA-Seq alignment files (described above) generated from HISAT2 using the assembly as the reference and TransDecoder [34] to generate an annotation file based on transcripts. Finally, the three respective annotation files were combined using EVidenceModeler (RRID:SCR_014659) [35]. After combining these gene structure predictions, we obtained 30,565 consensus protein-coding genes (Table 7). We annotated the genes using BLASTp searching against the NCBI nonredundant protein database (nr) and found that 78.3% of the predicted genes had at least one significant homologue (E-value < 1E-3), indicating that the gene structures were credible. We found that the majority of homologous proteins belonged to cucurbitaceous plants, such as cucumber and muskmelon (Fig. 3). Protein domain and gene ontology term annotations were performed using InterProScan 5 (RRID:SCR_005829, Table 7) [36]. In addition, genes annotated as SQEs, EPHs, CDSs, EPHs, CYP450s, and UGTs were compared with those in other Cucurbitaceae genomes, and we found that gene abundance in the five mogroside-related gene families were not significantly different among *S. grosvenorii, Cucumis sativus, Cucurbita moschata*, and *Cucurbita maxima* ([29], Table 8).

**Table 7:** Gene prediction and annotation

	RNA-Seq data-based	Ab initio	Homology-based	Integration	Annotation		
Weight	10	0.1	5	-	-		
Number of predicted genes	27,304	60,818	130,686	30,565	nr	IPR	GO
					23,936	19,684	14,966
Tools	HISAT2 StringTie TransDecoder	RepeatMasker AUGUSTUS	BLAST GeneWise	EVM	BLAST	InterProScan	

**Table 8:** Abundance analysis of the mogrosides synthesis related gene families

	*S. grosvenorii*	*C. sativus*	*C. moschata*	*C. maxima*
SQE	5 (5)	1	2	1
EPH	30 (8)	23	29	22
CYP450	276 (191)	213	289	234
UGT	156 (131)	124	137	121
CDS	1 (1)	1	2	3

The numbers quoted are the number of genes belonging to each gene family annotated in monk fruit genome version 1.

### Ortholog analysis

**Figure 4:**
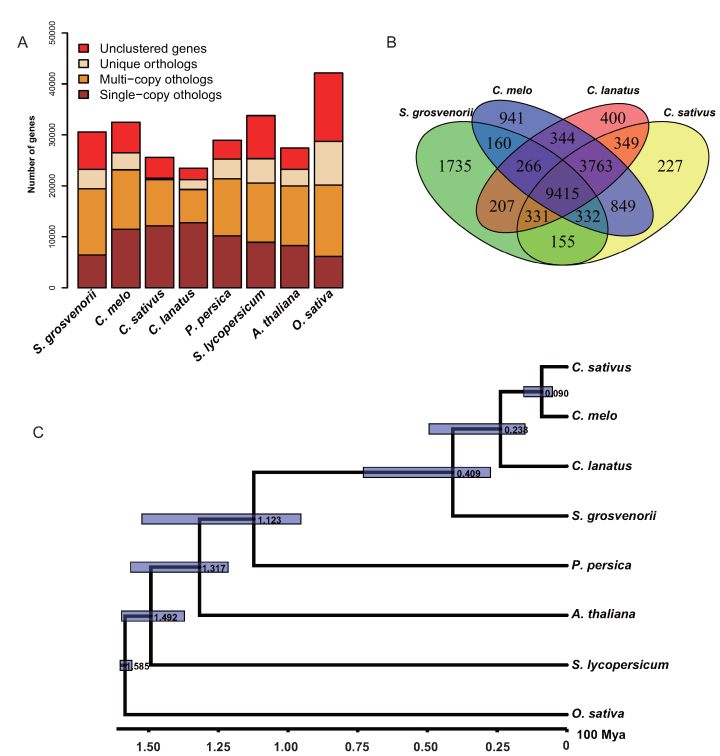
Comparative genome analysis of the *S. grosvenorii* genome. (**A**) Orthologue clustering analysis of the protein-coding genes in the *S. grosvenorii* genome. (**B**) Venn diagram showing shared and unique gene families among four cucurbit plant species. Numbers represent the number of gene families in unique or shared regions. (**C**) Phylogenetic tree and divergence time of *S. grosvenorii* and seven other plant species. The phylogenetic tree was generated from 834 single-copy orthologues using the maximum-likelihood method. The divergence time range is shown in blue blocks. The numbers beside the branching nodes are the predicted divergence time.

Gene family clustering analysis was accomplished using OrthoMCL (RRID:SCR_007839) [37] on protein sequences of *S. grosvenorii, C. sativus* (cucumber_ChineseLong_v2, [29]) [28], *Cucumis melo* (CM3.5.1, [29]) [38], *Citrullus lanatus* (watermelon_97 103_v1, [29]) [39], *Prunus persica* (Prunus_persica.prupe1_0, [40]) [41], *Solanum lycopersicum* (Solanum_lycopersicum.SL2.50, [40]) [42], *Arabidopsis thaliana* (Tair10, [43]) [44], and *Oryza sativa* (Oryza_sativa.IRGSP-1.0,^40^ ]) [45]. A total of 23,246 *S. grosvenorii* genes were clustered into 26,190 gene families, including 1,471 unique *S. grosvenorii* gene families (Fig. 4A). Compared to other cucurbitaceous plants, *S. grosvenorii* shares fewer gene families with relative species (Fig. 4B), indicating an earlier divergence time than *C. lanatus*. A total of 834 single-copy gene families were identified and selected to construct the phylogenetic tree using RAxML (RRID:SCR_006086) [46]. We used Muscle (RRID:SCR_011812) [47, 48] to align the orthologs, and the alignment was treated with Gblocks [49] with parameters of -t = p -b5 = h -b4 = 5 –b3 = 15 -d = y -n = y. The divergence time was estimated by MCMCtree [50]. Phylogenetic analysis showed that *S. grosvenorii* diverged from the Cucurbitaceae family approximately 40.95 million years ago (Fig. 4C).

### Transcriptomic analysis

**Figure 5:**
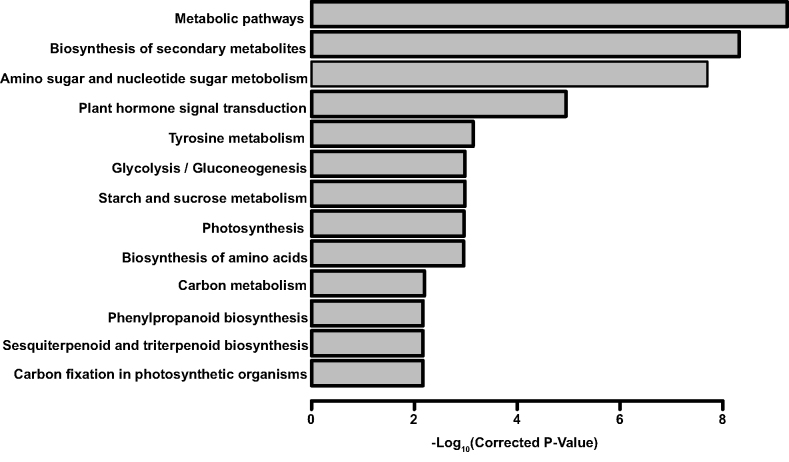
KEGG pathway enrichment analysis of candidate functional genes

Mogrosides are produced during fruit development in *S. grosvenorii* and are not found in vegetative tissues [8]. Thus, we performed an extensive transcriptomic analysis of early fruit at two stages (stage 1 sampled at 3 days after anthesis and stage 2 sampled at 20 days after anthesis) and of leaves to identify transcripts involved in mogroside synthesis in early fruit. Using the genome-wide annotation, RNA-Seq reads were mapped to the genome assembly, and read count tables were generated using HISAT2 and StringTie [33] for the next step of differential expression analysis. DESeq2 (RRID:SCR_000154) [51] was used to detect differential gene expression among L, F1, and F2 with the criteria of padj < 0.01 and |log2FoldChange| > 1. Genes that were up-regulated with fruit development were merged and used for KEGG pathway enrichment analysis with KOBAS (RRID:SCR_006350) [52]. Thirteen pathways were significantly enriched (corrected *P* < 0.01), and the most enriched pathways were related to metabolic pathways. In particular, the sesquiterpenoid and triterpenoid biosynthesis pathways were significantly enriched, indicating that genes involved in the biosynthesis of secondary metabolites, including mogrosides, perform their functions in the very early fruit (Fig. 5). Genes possibly related to mogrosides biosynthesis in early fruit according to the gene annotation were assigned to the mogrosides synthesis pathway (Fig. 2).

## Discussion

*S. grosvenorii* is an important herbal crop with multiple economic and pharmacological values. Mogrosides, the main effective components of *S. grosvenorii* fruit, are partial substitutes of sucrose because of its extremely sweet and noncaloric characteristics as more progress is made on molecular breeding and purification processes. Additionally, monk fruit could serve in contrast to other cucurbitaceous plant because of its earlier divergence from the common ancestor than some other well-studied cucurbits (cucumber, muskmelon), and it may be a new system for the investigation of plant sex determination. In the present study, we sequenced and assembled the second version of the monk fruit genome. With a great improvement in completeness and accuracy, the genome as well as the annotations will provide valuable resources and reference information for transcriptome assembly and novel gene discovery. These resources and further transcriptomics analysis of ripe fruit and young fruit will facilitate studies of the secondary metabolite synthesis pathways and monk fruit breeding.

## Availability of supporting data

The genomic and transcriptomic sequencing reads were deposited in the Genome Sequence Archive under accession number CRA000522 and ENA (European Nucleotide Archive) under accession numbers PRJEB23465, PRJEB23466, and PRJEB25737. Supporting data are also available in the *GigaScience* database, GigaDB [53].

**Abbreviations**

CDS: cucurbitadinenol synthase; CYP450: cytochrome P450; EPH: epoxide hydrolase; F1: fruit of 3 DAA; F2: fruit of 20 DAA; FL: female plants; L: leaves beside fruits; ML: male plants; PacBio: Pacific Biosciences; PE: paired-end; R: root; SMRT: single molecule real time sequencing; SQE: squalene epoxidase; UGT: UDP-glycosyl-transferase.

## Competing interests

The authors declare that they have no competing interests.

## Authors' contributions

X.W.D., B.C., H.H., and M.X. planned and coordinated the project. M.X. collected and grew the plant material. R.Y. and G.Z. collected the samples and performed experiments. Genome assembly, annotation, phylogenetic analysis, and manuscript writing were completed by X.H., M.X., H.H., and X.W.D.

## Supplementary Material

GIGA-D-17-00311_Original_Submission.pdf

GIGA-D-17-00311_Revision_1.pdf

GIGA-D-17-00311_Revision_2.pdf

Response_to_Reviewer_Comments_Original_Submission.pdf

Response_to_Reviewer_Comments_Revision_1.pdf

Reviewer_1_Report_(Original_Submission) -- John Hamilton 1/8/2018 Reviewed

Reviewer_2_Report_(Original_Submission) -- Aleksey Zimin 1/9/2018 Reviewed

Reviewer_2_Report_(Revision_1) -- Aleksey Zimin 4/6/2018 Reviewed
