# Supplementary material for: Improved de novo genome assembly and analysis of the Chinese cucurbit Siraitia grosvenorii, also known as monk fruit or luo-han-guo
Source: Gigascience. 2018 Jun 8;7(6):giy067. doi: 10.1093/gigascience/giy067 (PMC6007378; doi:10.1093/gigascience/giy067)

GigaScience

Improved de novo genome assembly and analysis of the Chinese cucurbit Siraitia grosvenorii, also known as monk fruit or luo-han-guo

--Manuscript Draft--

**Manuscript Number:**	GIGA-D-17-00311
**Full Title:**	Improved de novo genome assembly and analysis of the Chinese cucurbit Siraitia grosvenorii, also known as monk fruit or luo-han-guo
**Article Type:**	Data Note
**Funding Information:**	National Key R&D Program of China (2017YFA0503800) — Pro. Xing Wang Deng
**Abstract:**	Abstract<br>Backgroud: Luo-han-guo (Siraitia grosvenorii), also called monk fruit, is a member of the Cucurbitaceae family. To date, monk fruit is becoming a heated point of research for the pharmacological and economic potential of its non-caloric, extremely sweet components (mogrosides). It has also been commonly used in traditional Chinese medicine for the treatment of lung congestion, sore throat and constipation. Recently a single reference genome became available for monk fruit, assembled from 36.9 x genome coverage reads via Illumina sequencing platfroms. This genome assembly has a relatively short (34.2 kb) contig N50 length and lacks an integrated annotation. These drawbacks make it difficult to use as a reference in assembling transcriptomes and discovering novel functional genes.<br><br>Findings: Here we offer a new high-quality draft of S. grosvenorii genome assembled using 31 Gb (~ 73.8 x) long single molecule real time sequencing (SMRT) reads. The final genome assembly is approximately 467.1 Mb, with contig N50 length of 556,347 bp, representing a 12.7 fold improvement. We further annotated 237.3 Mb of repetitive sequence and 21,731 consensus protein coding genes with combined evidence. Phylogenetic analysis showed that S. grosvenorii diverged from members of cucurbitaceae family approximately 38.22 million years ago. With comprehensive transcriptomic analysis and differential expression test, we identified 825 candidate functional transcripts involved in mogrosides biosynthesis.<br><br>Conclusions: The availability of this new monk fruit genome assembly as well as candidate transcirpts will facilitate the discovery of new functional genes and genetic improvement of monk fruit.<br><br>Keywords: Siraitia grosvenorii, Monk fruit, PacBio sequencing, Ortholog analysis, RNA-seq, Mogrosides biosynthesis
**Corresponding Author:**	Hang He<br><br>beijing, Beijing CHINA
**Corresponding Author Secondary Information:**	
**Corresponding Author's Institution:**	
**Corresponding Author's Secondary Institution:**	
**First Author:**	Mian Xia
**First Author Secondary Information:**	
**Order of Authors:**	Mian Xia<br>Xue Han<br>Hang He<br>Renbo Yu

Powered by Editorial Manager® and ProduXion Manager® from Aries Systems Corporation

	Gang Zhen
	Xiping Jia
	Beijiu Cheng
	Xing Wang Deng
**Order of Authors Secondary Information:**	
**Opposed Reviewers:**	
**Additional Information:**	
**Question**	**Response**
Are you submitting this manuscript to a special series or article collection?	No
**Experimental design and statistics**<br><br>Full details of the experimental design and statistical methods used should be given in the Methods section, as detailed in our Minimum Standards Reporting Checklist. Information essential to interpreting the data presented should be made available in the figure legends.<br><br>Have you included all the information requested in your manuscript?	Yes
**Resources**<br><br>A description of all resources used, including antibodies, cell lines, animals and software tools, with enough information to allow them to be uniquely identified, should be included in the Methods section. Authors are strongly encouraged to cite Research Resource Identifiers (RRIDs) for antibodies, model organisms and tools, where possible.<br><br>Have you included the information requested as detailed in our Minimum Standards Reporting Checklist?	Yes
**Availability of data and materials**<br><br>All datasets and code on which the conclusions of the paper rely must be either included in your submission or deposited in publicly available repositories (where available and ethically appropriate), referencing such data using a unique identifier in the references and in the "Availability of Data and Materials" section of your manuscript.	Yes

Have you have met the above requirement as detailed in our Minimum Standards Reporting Checklist?	

Powered by Editorial Manager® and ProduXion Manager® from Aries Systems Corporation

Manuscript

Click here to download Manuscript monkfruit_manuscript_corrected.docx

Improved *de novo* genome assembly and analysis of the Chinese cucurbit *Siraitia grosvenorii*, also known as monk fruit or luo-han-guo

Mian Xia[1, †], Xue Han[2, †], Hang He[2, †], Renbo Yu[2], Gang Zhen[2], Xiping Jia[3], Beijiu Cheng[1,*] and Xing Wang Deng[2,*]

[1]Key Laboratory of Crop biology of Anhui Province, Anhui Agricultural University, Hefei, China

[2]School of Advanced Agriculture Sciences and School of Life Sciences, State Key Laboratory of Protein and Plant Gene Research, Peking University, Beijing 100871, China.

[3]National Demonstration Area of Modern Agriculture in Cangxi, Sichuan Province, China

[*]Correspondence: Xing Wang Deng (deng@pku.edu.cn), Beijiu Cheng (cbj@ahau.edu.cn)

[†]Theses authors contributed equally to this article.

Abstract

Backgroud: Luo-han-guo (*Siraitia grosvenorii*), also called monk fruit, is a member of the Cucurbitaceae family. To date, monk fruit is becoming a heated point of research for the pharmacological and economic potential of its non-caloric, extremely sweet components (mogrosides). It has also been commonly used in traditional Chinese medicine for the treatment of lung congestion, sore throat and constipation. Recently a single reference genome became available for monk fruit, assembled from 36.9 x genome coverage reads via Illumina sequencing platfroms. This genome assembly has a relatively short (34.2 kb) contig N50 length and lacks an integrated annotation. These drawbacks make it difficult to use as a reference in assembling

transcriptomes and discovering novel functional genes.

Findings: Here we offer a new high-quality draft of *S. grosvenorii* genome assembled using 31 Gb (~ 73.8 x) long single molecule real time sequencing (SMRT) reads. The final genome assembly is approximately 467.1 Mb, with contig N50 length of 556,347 bp, representing a 12.7 fold improvement. We further annotated 237.3 Mb of repetitive sequence and 21,731 consensus protein coding genes with combined evidence. Phylogenetic analysis showed that *S. grosvenorii* diverged from members of cucurbitaceae family approximately 38.22 million years ago. With comprehensive transcriptomic analysis and differential expression test, we identified 825 candidate functional transcripts involved in mogrosides biosynthesis.

Conclusions: The availability of this new monk fruit genome assembly as well as candidate transcirpts will facilitate the discovery of new functional genes and genetic improvement of monk fruit.

Keywords: *Siraitia grosvenorii*, Monk fruit, PacBio sequencing, Ortholog analysis, RNA-seq, Mogrosides biosynthesis

Data description

Introduction

Siraitia grosvenorii (luo-han-guo or monk fruit, NCBI Taxonomy ID: 190515) is an herbaceous perennial native to southern China and is a famous specialty in Guilin city, Guangxi Province of China (Figure 1)[1]. On top of being used as a natural sweetener, *S. grosvenorii* has been used in China as a folk remedy for the treatment of lung congestion, sore throat and constipation for

hundreds of years[2]. The ripe fruit of *S. grosvenorii* contains mogrosides, which have become a popular research topic due to their pharmacological characteristics, including putative anti-cancer properties [3]. Additionally, mogrosides are purified and used as a non-caloric, non-sugar sweetener in the United States and Japan, as they are estimated to be approximately 300 times as sweet as sucrose [1,4]. To date, *S. grosvenorii* fruit has been shown to have the following extra effects of antitussive, anti-asthmatic, anti-oxidation, liver-protection, glucose-lowering, immunoregulation, and shown as containing triterpenoids, flavonoids, vitamins, proteins, saccharides, and a volatile oil [5,6]. Monk fruit products have been approved as dietary supplements in Japan, the US, New Zealand and Australia [2,7].

The biosynthesis pathway of mogrosides has been extensively studied and several genes have been identified [8-11]. Squalene is thought to be the initial substrate and precursor for triterpenoid and sterol biosynthesis. Squalene epoxidases (SQE) perform expoxidation, which creates squalene or oxidosqualene, and cucurbitadinenol synthase (CDS) cyclizes oxidosqualene to form the cucurbitadienol triterpenoid skeleton, which is a distinct step in phytosterol biosynthesis [12]. Epoxide hydrolases (EPH) and cytochrome P450s (CYP450) further oxidize cucurbitadienols to produce mogrol, which is glycosylated by UDP-glycosyl-transferases (UGT) to form mogroside V (Figure 2).

The genome of *S. grovenorii* was first published in 2016, served the purpose of identifying the genomic organization of the gene families of interest, but not as the reference in their transcripotome assembly and gene families identification[8]. Although the fact that the first draft genome assembly was useful resources, some improvements are still necessary, including

improving the continuity and completeness, genome assembly assessment, annotation of genes and repetitive regions, and other genomic features analysis. With average read length now exceeding 10 kb, SMRT sequencing technology from Pacific Biosciences (PacBio) has the potential to significantly improve genome assembly quality [13]. Therefore, we *de novo* assembled a high-quality genome draft of *S. grosvenorii* using high coverage of PacBio long reads and applied extensive genomic and transcriptomic analysis. This new assembly, annotation and other genomic features studied below will serve as a valuable resource for investigating economic and pharmacological characters and assisting molecular breeding of monk fruit.

Library construction and sequencing of single-molecule long reads

20 μg genomic DNA was extracted from seedlings of *S. grosvenorii* (variety Qingpiguo) using a modified CTAB method [14] to construct 2 libraries with an insert size of 20 kb, which were introduced from the Yongfu District (Guangxi Province, China) and planted in Cangxi County (Sichuan Province, China). Sequencing of *S. grosvenorii* was performed using the Pacbio RSII platform (Pacfic Biosciences; USA) and generated 31 Gb (~ 73.8 x) of data from 44 SMRT cells, with an average subread length of 7.7 kb and read quality of 82 % after filtering low-quality bases and adapters (Table 1).

RNA isolation and sequencing

Fresh roots, leaves and early fruit of *S. grosvenorii* were sampled in the garden of Cangxi County. All samples were stored at -80 °Cafter treated immediately with liquid nitrogen. Total RNA was

isolated from (1) leaf of female plants (FL), (2) leaves of male plants (ML), (3) leaves beside fruits (L), (4) roots(R), (5) fruit of 3 DAA (F1) and (6) fruit of 20 DAA (F2) using Qiagen RNeasy Plant Mini Kits (Qiagen). Paired-end libraries (PE150 with insertion size of 350 bp) were constructed and subsequently sequenced via Illumina HiSeq X-Ten platform (Illumina; CA, USA).

Table 1 SMRT reads used for genome assembly

**Statistics**	**Length (bp)**
Total raw data	31 G
Mean length of raw reads	11 K
N50 of raw reads	15,754
Mean length of subreads	7.7 K
N50 of subreads	11,898

Subreads: reads without adapters and low-quality bases.

Genome assembly

Initial correction of long reads was carried out using FALCON [15] with length_cutoff = 5000 according to the distribution of read length and -B15, -s400 to cut reads into blocks of 400Mb and aligned 15 blocks to another one at the same time. The longest 25 x corrected reads was extracted with Perl scripts and assembled by mecat2canu command of MECAT [16] with GenomeSize=420000000 estimated in previous study. This led to a new genome assembly of 467 Mb with a contig N50 size of 434,684 bp (Table 2). This genome size was slightly larger than the estimated 420 Mb [8], which was probably due to the high genome heterozygosity. The assembly produced 4,128 contigs, 609 of which were over 100 kb long. Genome scaffolding was processed

respectively using SSPACE-LongRead [17] with all the SMRT long read sequences and AGUTI [18] with paired-end RNA-seq reads of root, leaf and fruit (Table 2). Compared to the preliminary draft of the published *Siraitia* genome, the contiguity was improved more than ~12.7 times.

Genome assessment

We estimated the completeness of the assembly by using Benchmarking Universal Single-Copy Orthologues (BUSCO v2, RRID:SCR_015008) [19] analysis. Of the 1,440 orthologues identified in plants, 1,167 were found in the genome assembly, including 877 in single copy and 290 in multi-copy (Table 3).

In addition, we used RNA-seq data from different organs to assess the sequence quality. The assembly was mapped by all the 15 RNA-seq raw data using HISAT2 (RRID:SCR_015530)[20] and overall alignment rate of each data was used as rough estimation of sequence quality. Then the alignment files was manipulated by SAMtools (RRID:SCR_002105) [21] and only unique mappings (mapping quality = 60) were retained to call SNP with Genome Analysis Toolkit (GATK, RRID:SCR_001876) [22] pipeline. GATK VariantFiltration program was used to filter out low quality variations with the following expression : QD < 2.0 || ReadPosRankSun < - 8.0 || FS > 60.0 || QUAL < 50 || DP < 5. Coverage of each uniq alignment file was scanned using Qualimap 2 [23] and error rate was calculated as the ration of double variation (1/1 and 1/2) number and covered genome size. The overall alignment rates of reads in most samples were over 80% (Table 4), and the average base error rate was estimated at 0.07%, which suggests a high-quality assembly (Table 5).

Table 2 Metrics of *de novo S. grosvenorii* genome assembly

Statistics	Contig	Scaffold (SSPACE_LongRead)	Scaffold (AGOUTI)
Total number	4,128	3,429	4,053
Total length (bp)	467,072,951	468,956,921	467,147,951
N50 length (bp)	433,684	549,749	456,454
N90 length (bp)	36,820	41,649	37,010
Max length (bp)	7,657,852	7,657,852	7,657,852
GC content (%)	33.57	33.57	33.57
N length (bp)	0	1,883,970	75,000

Table 3 Summarized benchmarks of the BUSCO assessment.

	Monk fruit (%)
Complete BUSCOs	81.0
Complete and single-copy	60.9
Complete and duplicated	20.1
Partial	5.1
Missing	13.9

Repeat annotation

We scanned the genome using RepeatMasker (RRID:SCR_012954) [24] with Repbase [25] and a de novo repeat database constructed with RepeatModeler (RRID:SCR_015027) [26]. We

identified 237 Mb (50.8% of the assembled genome) as repetitive elements, which was slightly higher than the 42.8% of *Momordica charantia* [27] and much higher than the 28.2% of *Cucumis sativus* [28]. We further classified the repetitive regions and found that the vast majority was interspersed repeats. Among them, the main subtypes were unclassified repeats and long terminal repeats (LTRs), and Copia (30.7 Mb, 6.6% of the genome) and Gypsy (41.6 Mb, 8.9% of the genome) LTRs were the most abundant. Compared to cucumber, the genome enlargement in monk fruit and bitter gourd was likely driven by the expansion of interspersed repeats (Table 6).

Table 4 Quality evaluation of the draft genome with overall alignment rate

Sample	Overall alignment rate
FL-1	87.06%
FL-2	84.84%
FL-3	82.94%
ML-1	87.08%
ML-2	87.17%
ML-3	82.54%
L-1	83.42%
L-2	84.42%
R-1	79.30%
R-2	82.03%
R-3	82.33%
F1-1	82.25%
F1-2	89.40%
F2-1	84.82%
F2-2	85.25%

Table 5 Genome base accuracy estimated using RNA-seq reads

Sample	Coverage	Variation				Error rate
		0/1	1/1	1/2	Total	
FL-1	15.3%	9,489	9,355	489	19,333	1.4E-4
FL-2	13.6%	60,145	65,778	2,897	128,820	1.1E-3
FL-3	15.4%	74,724	83,290	3,473	161,487	1.2E-3
ML-1	16.3%	24,003	28,475	940	53,418	3.9E-4
ML-2	16.6%	35,480	46,177	1,301	82,958	6.1E-4
ML-3	16.7%	44,176	63,115	1,513	108,804	8.3E-4
L-1	16.0%	48,632	50,938	2,022	101,592	7.1E-4
L-2	15.2%	57,994	55,795	2,533	116,322	8.2E-4
R-1	11.5%	51,240	51,216	2,114	104,570	9.9E-4
R-2	9.0%	43,058	37,967	1,886	82,911	9.4E-4
R-3	11.3%	5,939	5,271	283	11,507	1.1E-4
F1-1	9.3%	31,531	33,663	1,292	66,486	8.1E-4
F1-2	16.9%	20,019	19,083	869	39,998	2.5E-4
F2-1	10.6%	47,261	41,679	2,100	91,040	8.9E-4
F2-2	11.8%	52,576	48,655	2,279	103,510	9.2E-4

High-quality genome criteria: 1E-4.

FL: female leaf, ML: male leaf, L: leaf, R: root, F1: fruit stage 1, F2: fruit stage 2.

0: genotype that is identical to the reference, 1,2: genotype that is different from the reference.

Gene annotation

To generate gene models, the *S. grosvenorii* genome sequences were subjected to 3 gene

prediction pipelines including homology-based, de novo and RNA-seq data-based prediction. First, we aligned the assembly sequences to cucumber protein sequences downloaded from cucurbit database using BLASTX and merged the hits if intervals of 2 hits was less than 6,000 bp [29]. The merged sequences was extracted and further scanned for protein coding gene structures by GeneWise (RRID:SCR_015054, https://www.ebi.ac.uk/~birney/wise2/). Second, we de novo predicted protein coding genes using AUGUSTUS (RRID:SCR_008417) [30] with a repeat masked genome, while repeat masking was done by RepeatMasker. Third, we used StringTie [31] assemble 15 RNA-seq alignment files (described above) generated from Hisat2 to transcriptome with the assembly as reference, and TransDecoder (https://github.com/TransDecoder/TransDecoder) to identify coding regions based on transcripts. In the end, three respective annotation files were combined using EVidenceModeler (EVM, RRID:SCR_014659) [32]. After combining these gene structure predictions, we obtained 21,731 consensus protein-coding genes (Table 7). We annotated the genes using BLASTX with the non-redundant database and found that 84.7% of the predicted genes had at least one significant homologue, indicating that the gene structures were credible. We found that 10,678 of the homologous proteins belonged to cucurbitaceous plants, such as cucumber (Chinese Long v2) and muskmelon (Figure 3). Protein domain annotations and gene ontology (GO) terms were assigned using InterProScan 5 (RRID:SCR_005829, Table 7) [33].

Synteny analysis

We compared the monk fruit genome to the cucumber genome using integrated genome

annotation and synteny mapping of protein-coding sequences with the SyMap 4.2 program [34]. Synteny blocks were observed in 1,992 of 4,128 contigs and were defined as regions consisting of more than seven anchors between two species [26]. These anchored contigs comprised 76.5% of the genome, whereas the anchor region covered 9.5% of the monk fruit genome and 17.4% of the cucumber genome. Thus, monk fruit and cucumber share a large number of similar genes, even though their genome sizes differ greatly.

Table 6 Repeat annotation of the *S. grosvenorii* genome

**Repeat Classification**		***Siraitia grosvenorii***		***Momordica charantia***		***Cucumis sativus***	
		Length (bp)	Content	Length (bp)	Content	Length (bp)	Content
Interspersed repeats	SINEs	0	0.00%	0	0.00%	0	0.00%
	LINEs	10,114,693	2.17%	5,183,926	1.82%	2,397,830	1.22%
	LTR	73,041,961	15.64%	34,217,647	11.98%	8,253,090	4.18%
	DNA elements	9,070,191	1.94%	3,460,431	1.21%	2,777,943	1.41%
	Unclassified	139,015,592	29.76%	75,056,338	26.28%	37,539,553	19.03%
	Total	231,242,473	49.51%	117,918,342	41.29%	50,967,966	25.84%
Simple repeats		5,447,789	1.17%	3,451,508	1.21%	3,547,474	1.80%
Low complexity		1,514,238	0.32%	958,289	0.34%	1,095,406	0.56%
Total		237,342,400	50.81%	122,111,538	42.75%	55,540,243	28.15%

Ortholog analysis

Gene family clustering analysis was accomplished using OrthoMCL (RRID:SCR_007839) [35] on protein sequences of *S. grosvenorii*, *C. sativus* (cucumber_ChineseLong_v2, http://cucurbitgenomics.org/) [27], *Cucumis melo* (CM3.5.1, http://cucurbitgenomics.org/) [36], *Citrullus lanatus* (watermelon_97103_v1, http://cucurbitgenomics.org/) [37], *Prunus persica* (Prunus_persica.prupe1_0, https://plants.ensembl.org/) [38], *Glycine max* (Glycine_max_V1.0, http://plants.ensembl.org/) [39] and *Arabidopsis thaliana* (Tair10, http://Arabidopsis.org/) [40]. A total of 15,576 *S. grosvenorii* genes were clustered into 8,543 gene families, including 4,178 unique S. *grosvenorii* genes (Figure 4A). Compared to other cucurbitaceous plants, *S. grosvenorii* shares fewer gene families (Figure 4B), indicating an earlier divergence time than *C. lanatus* . 229 single-copy gene families were identified, and 164 groups high-quality orthologs among them were selected to construct the phylogenetic tree using RAxML (RRID:SCR_006086) [41]. We used Muscle (RRID:SCR_011812, https://www.ebi.ac.uk/Tools/msa/muscle/) [42] to align the orthologs and the alignment was treated with Gblocks [43] with parameters of -t=p -b5=h -b4=5 -d=y -n=y. The divergence time was estimated by MCMCtree [44]. Phylogenetic analysis showed that *S. grosvenorii* diverged from the cucurbitaceae family approximately 38.22 million years ago (Figure 4C). In addition, we annotated the orthologue groups belonging to SQEs, CDSs, EPHs, CYP450s, and UGTs, and we found that gene abundance in the 5 mogroside-related gene families was not significantly different (Table 8).

Table 7 Gene prediction and annotation

	RNA-seq data-based	Ab initio	Homology-based	Integration	Annotation		
**Weight**	20	1	1	-	-		
					nr	IPR	GO
**Number of predicted genes**	27,229	76,804	261,439	21,731	18,411	12,305	8,626
**Tools**	HISAT2<br>StringTie<br>TransDecoder	RepeatMasker<br>AUGUSTUS	BLAST<br>GeneWise	EVM	BLAST	InterProScan	

Table 8 Abundance analysis of the mogroside synthesis related gene families

	Siraitia grosvenorii	***Cucumis sativus***	***Cucumis melo***	***Citrullus lanatus***
SQE	4 (5)	4	4	5
EPH	24 (8)	33	35	29
CYP450	149 (191)	158	185	168
UGT	57 (131)	60	71	74
CDS	13 (1)	5	9	8

Transcriptomic analysis

Mogrosides are produced during fruit development in *S. grosvenorii* and are not found in vegetative tissues [8]. Thus, we performed an extensive transcriptomic analysis of early fruit at 2 stages (stage 1 sampled 3 days after anthesis and stage 2 sampled at 20 days after anthesis) and

of leaves to identify transcripts involved in mogroside synthesis. Using the genome-wide annotation, RNA-seq reads were mapped to the genome assembly and 77,844 transcripts were assembled for differential expression analysis using Hisat2. Deseq2 (RRID:SCR_000154) [45] was used to detect differential expression transcripts (DET) among leaves (L), fruits of 3 DAA (F1) and fruits of 20 DDA(F2) with the criteria of padj < 0.1. Transcripts that were significantly highly expressed in fruit were merged (Figure 5A), and 825 were found to increase from leaves to fruit in stages 1 and 2. These were chosen as functional candidate transcripts for KEGG pathway enrichment analysis using KOBAS (RRID:SCR_006350) [46]. Twelve pathways were significantly enriched (Corrected P-value < 0.05), and the most enriched pathways were related to secondary metabolites. In particular, the sesquiterpenoid and triterpenoid biosynthesis pathways were significantly enriched (Figure 5B). We found 825 functional transcript candidates with similarity to proteins in 5 mogroside-related cucurbit gene families. We used BLASTX to detect 0 SQE, 5 CDS, 6 EPH, 19 CYP 450 and 6 UGT homologues, which were assigned to the mogrosides synthesis pathway (Figure 2). All transcripts were queried against the non-redundant database and annotated with the Blast2GO (RRID:SCR_005828) [47] platform. In addition to the 36 transcripts of the five gene families, 64 transcription factors, 72 transporters and 331 other enzymes were detected through annotation (Figure 2). These transcripts are possibly novel genes related to mogroside synthesis.

Discussion

Siraitia grosvenorii is an important herbal crop with multiple economic and pharmacological

values. Mogrosides, the main effective components of *S. grosvenorii* fruit, will be partial substitutes of sucrose for its extreme sweet and non-caloric characters as more and more progress has been making on molecular breeding and purification process. Additionally, monk fruit could serve as the contrast of other cucurbitaceous plant as its earlier divergence from the common ancestor than some other well-studied cucurbits (cucumber, muskmelon et al.) and a new system for the investigation of plant sex determination. In the present study, we sequenced and assembled the second version of monk fruit genome. With the great improvement of completeness and accuracy, the genome as well as the annotations will provide valuable resources and reference information for transcriptomes assembly and novel gene discovery as we did above. With the resources and further transcriptomic analysis of ripe fruit and young fruit will facilitate studies of the mogrosides synthesis pathway and monk fruit breeding.

Availability of supporting data

The genomic and transcriptomic sequencing reads have been deposited in the Genome Sequence Archive (GSA) under the Accession of CRA000522 and ENA (European Nucleotide Archive) under the Accession number of PRJEB23465, PRJEB23466. Supporting data are also available in the GigaScience database, GigaDB.

ACKNOWLEDGMENTS

This research was supported by National Key R&D Program of China (2017YFA0503800) to X.W.D. and in part by National Demonstration Area of Modern Agriculture in Cangxi, Sichuan

Province, China.

Author's contribution

XWD, BC, HH, and MX planned and coordinated the project. MX collected and grew the plant material. RY and GZ collected the samples and performed experiments. Genome assembly, annotation, phylogenetic analysis and manuscript writing were completed by XH, MX, HH and XWD.

Competeing interests

The authors declare that they have no competing interests.

Reference

1. Zhang JS, Dai LH, Yang JG, Liu C, Men Y, Zeng Y, et al. Oxidation of cucurbitadienol catalyzed by CYP87D18 in the biosynthesis of mogrosides from *Siraitia grosvenorii.* Plant Cell Physiol. 2016;57:1000-1007.
2. Li C, Lin LM, Sui F, Wang ZM, Huo HR, Dai L, et al. Chemistry and pharmacology of Siraitia grosvenorii: A review. Chinese Journal of Natural Medicines. 2014;12:89-102.
3. Liu C, Dai LH, Dou DQ, Ma LQ, Sun YX. A natural food sweetener with anti-pancreatic cancer properties. Oncogenesis. 2016;5:e217.
4. Nie RL. The decadal progress of triterpene saponins from *Cucurbitaceae* (1980–1992). Acta Bot Yunnan 1994;16:201–208.

5. Wang Q, Qin HH, Wang W, Qiu SP. The pharmacological research progress of *Siraitia grosvenorii.* J Guangxi Tradit Chin Med Univ. 2010;13:75-76.

6. Zhang H, Li XH. Research progress on chemical compositions of Fructus Momordicae. J Anhui Agri Sci. 2011;39:4555-4556, 4559.

7. Pawar RS, Krynitsky AJ, Rader JI. Sweeteners from plants--with emphasis on Stevia rebaudiana (Bertoni) and Siraitia grosvenorii (Swingle). Anal Bioanal Chem. 2013 ;405:4397-407.

8. Itkin M, Davidovich-Rikanati R, Cohen S, Portnoy V, Doron-Faigenboim A, Oren E, et al. The biosynthetic pathway of the nonsugar, high-intensity sweetener mogroside V from *Siraitia grosvenorii*. Proc Natl Acad Sci U S A. 2016;113:E7619-E7628.

9. Dai LH, Liu C, Zhu YM, Zhang JS, Men Y, Zeng Y, et al. Functional characterization of cucurbitadienol synthase and triterpene glycosyltransferase involved iin biosynthesis of mogrosides from *Siraitia grosvenorii*. Plant Cell Physiol. 2015;56: 1172-1182.

10. Zhang JS, Dai LH, Yang JG, Liu C, Men Y, Zeng Y, et al. Oxidation of cucurbitadienol catalyzed by CYP87D18 in the biosynthesis of mogrosides from *Siraitia grosvenorii.* Plant Cell Physiol. 2016;57:1000-1007.

11. Tang Q, Ma XJ, Mo CM, Wilson WI, Song C, Zhao H, et al. An efficient approach to finding *Siraitia grosvenorii* triterpene biosynthetic genes by RNA-seq and digital gene expression analysis. BMC Genomics. 2011;12: 343.

12. Shibuya M, Adachi S, Ebizuka Y. Cucurbitadienol synthase, the first committed enzyme for cucurbitacin biosynthesis, is a distinct enzyme from cycloartenol synthase for phytosterol

biosynthesis. Tetrahedron. 2004;60: 6995–7003.

13. Zimin AV, Stevens KA, Crepeau MW, Puiu D, Wegrzyn JL, Yorke JA, et al. An improved assembly of the loblolly pine mega-genome using long-read single-molecule sequencing. Gigascience. 2017;6:1-4.

14. Porebski S, Bailey LG, Baum BR. Modification of a CTAB DNA extraction protocol for plants containing high polysaccharide and polyphenol components. Plant Mol Biol Rep. 1997;15:8-15.

15. Chin CS, Peluso P, Sedlazeck FJ, Nattestad M, Concepcion GT, Clum A, et al. Phased diploid genome assembly with single-molecule real-time sequencing. Nat Methods. 2016;13:1050-1054.

16. Xiao CL, Chen Y, Xie SQ, Chen KN, Wang Y, Han Y, et al. MECAT: fast mapping, error correction, and de novo assembly for single-molecule sequencing reads. Nat Methods. 2017;14:1072-1074.

17. Boetzer M, Pirovano W. SSPACE-LongRead: scaffolding bacterial draft genomes using long read sequence information. BMC Bioinformatics. 2014;15:211.

18. Zhang SV, Zhuo L, Hahn MW. AGOUTI: improving genome assembly and annotation using transcriptome data. GigaScience. 2016;5:31.

19. Simão FA, Waterhouse RM, Ioannidis P, Kriventseva EV, Zdobnov EM. BUSCO: assessing genome assembly and annotation completeness with single-copy orthologs. Bioinformatics. 2015;31:3210-2.

20. Kim D, Langmead B, Salzberg SL. HISAT: a fast spliced aligner with low memory requirements. Nat Methods. 2015;12:357-60.

21. Li H, Handsaker B, Wysoker A, Fennell T, Ruan J, Homer N, et al. The Sequence alignment/map (SAM) format and SAMtools. Bioinformatics. 2009;25:2078-9.

22. McKenna A, Hanna M, Banks E, Sivachenko A, Cibulskis K, Kernytsky A, et al. The Genome Analysis Toolkit: a MapReduce framework for analyzing next-generation DNA sequencing data. Genome Res. 2010;20:1297-303.

23. Okonechnikov K, Conesa A, García-Alcalde F. Qualimap 2: advanced multi-sample quality control for high-throughput sequencing data. Bioinformatics. 2016;32:292-4.

24. Tarailo-Graovac M, Chen N. Using RepeatMasker to identify repetitive elements in genomic sequences. Curr Protoc Bioinformatics. 2009;3:4–14.

25. Visser M, Van der Walt AP, Maree HJ, Rees DJ G, Burger JT. Extending the sRNAome of apple by next-generation sequencing. PLoS one. 2014;9:e95782.

26. Smit A, Hubley R. RepeatModeler Open-1.0.8, 2008; http://www.repeatmasker. org/RepeatModeler.html.

27. Urasaki N, Takagi H, Natsume S, Uemura A, Taniai, N, Miyagi N, et al. Draft genome sequence of bitter gourd (*Momordica charantia*), a vegetable and medicinal plant in tropical and subtropical regions. DNA Res. 2016;24:51-58.

28. Huang S, Li R, Zhang Z, Li L, Gu X, Fan W, et al. The genome of the cucumber, *Cucumis sativus* L. Nat Genet. 2009;41:1275-81.

29. Gupta V, Estrada AD, Blakley I, Reid R, Patel K, Meyer MD, et al.

RNA-Seq analysis and annotation of a draft blueberry genome assembly identifies candidate genes involved in fruit ripening, biosynthesis of bioactive compounds, and stage-specific alternative splicing. Gigascience. 2015; 4:5.

30. Stanke M, Tzvetkova A, Morgenstern B. AUGUSTUS at EGASP: using EST, protein and genomic alignments for improved gene prediction in the human genome. Genome Biol. 2006;7:S11.1-8.

31. Pertea M, Pertea GM, Antonescu CM, Chang TC, Mendell JT, Salzberg SL. StringTie enables improved reconstruction of a transcriptome from RNA-seq reads. Nat Biotechnol. 2015;33:290-5.

32. Haas BJ, Salzberg SL, Zhu W, Pertea M, Allen JE, Orvis J, et al. Automated eukaryotic gene structure annotation using EVidenceModeler and the Program to Assemble Spliced Alignments. Genome Biol. 2008;9:R7.

33. Quevillon E, Silventoinen V, Pillai S, Harte N, Mulder N, Apweiler R, et al. InterProScan: protein domains identifier. Nucleic Acids Res. 2005;33:W116-20.

34. Soderlund C, Bomhoff M, Nelson WM. SyMAP v3.4: a turnkey synteny system with application to plant genomes. Nucleic Acids Res. 2011;39:e68.

35. Li L, Stoeckert CJJr, Roos DS. OrthoMCL: identification of ortholog groups for eukaryotic genomes. Genome Res. 2003;13:2178-89.

36. Garcia-Mas J, Benjak A, Sanseverino W, Bourgeois M, Mir G, González VM, et al. The genome of melon (Cucumis melo L.). Proc Natl Acad Sci U S A. 2012;109:11872-7.

37. Guo S, Zhang J, Sun H, Salse J, Lucas WJ, Zhang H, et al. The draft genome of watermelon (Citrullus lanatus) and resequencing of 20 diverse accessions. Nat Genet. 2013;45:51-8.

38. International Peach Genome Initiative, Verde I, Abbott AG, Scalabrin S, Jung S, Shu S, et al. The high-quality draft genome of peach (Prunus persica) identifies unique patterns of genetic diversity, domestication and genome evolution. Nat Genet. 2013;45:487-94.

39. Schmutz J, Cannon SB, Schlueter J, Ma J, Mitros T, Nelson W, et al. Genome sequence of the palaeopolyploid soybean. Nature. 2010;463:178-83.

40. Lamesch P, Berardini TZ, Li D, Swarbreck D, Wilks C, Sasidharan R, et al. The Arabidopsis Information Resource (TAIR): improved gene annotation and new tools. Nucleic Acids Res. 2012;40:D1202-10.

41. Stamatakis A. RAxML version 8: a tool for phylogenetic analysis and post-analysis of large phylogenies. Bioinformatics. 2014;30:1312-3.

42. Edgar RC. MUSCLE: multiple sequence alignment with high accuracy and high throughput. Nucleic Acids Res. 2004;32:1792-7.

43. Talavera G, Castresana J. Improvement of phylogenies after removing divergent and ambiguously aligned blocks from protein sequence alignments. Syst Biol. 2007;56:564-77.

44. Battistuzzi FU, Billing-Ross P, Paliwal A, Kumar S. Fast and slow implementations of relaxed-clock methods show similar patterns of accuracy in estimating divergence times. Mol Biol Evol. 2011;28:2439-42.

45. Love MI, Huber W, Anders S. Moderated estimation of fold change and dispersion for RNA-seq data with DESeq2. Genome Biol. 2014;15:550.
46. Xie C, Mao X, Huang J, Ding Y, Wu J, Dong S, et al. KOBAS 2.0: a web server for annotation and identification of enriched pathways and diseases. Nucleic Acids Res. 2011;39:W316-22.
47. Conesa A, Götz S, García-Gómez JM, Terol J, Talón M, Robles M. Blast2GO: a universal tool for annotation, visualization and analysis in functional genomics research. Bioinformatics. 2005;21:3674-6.

Figure legends

Figure 1 Morphological character of the fruit of *S. grosvenorii (A),* vertical section of fruit of *S. grosvenorii* (B), horizontal section of fruit of *S. grosvenorii* (C) and seeds (D). Size bar, 1 cm.

Figure 2 Candidate transcripts involved in mogrosides biosynthesis pathway. Candidate functional transcripts were annotated as homologues including enzymes, transcription factors and transporters, which were selected and assigned to mogrosides biosynthesis pathway.

Figure 3 Number of best-matching proteins for each predicted *S. grosvenorii* gene by species.

Figure 4 Comparative genome analysis of the *S. grosvenorii* genome. (A)Orthologue clustering analysis of the protein-coding genes in the *S. grosvenorii* genome. (B) Venn diagram showing shared and unique gene families among four cucurbit plant species. Numbers represent the number of gene families in unique or shared regions. (C) Phylogenetic tree and divergence time of *S. grosvenorii* and 6 other plant species. The phylogenetic tree was generated from 164 single-copy orthologues using the Maximum-likelihood method. The divergence time range is

shown in the blue blocks. The numbers beside the branching nodes are the predicted divergence time.

Figure 5 Expression pattern analysis of candidate functional transcripts involved in mogrosides synthesis pathway. (A) Expression heatmap of significantly highly expressed transcripts in fruit. Transcripts that were significantly highly expressed in fruit stage 1 (Fruit 1) or fruit stage 2 (Fruit 2) compared to those in leaves were merged and classified according to their expression. Only transcripts that belong to increasing expression patterns (red stars) were chosen as candidate functional transcripts for further analysis. (B) KEGG pathway enrichment analysis of candidate functional transcripts.

Figure 1

Click here to download Figure figure1-ps1.pdf

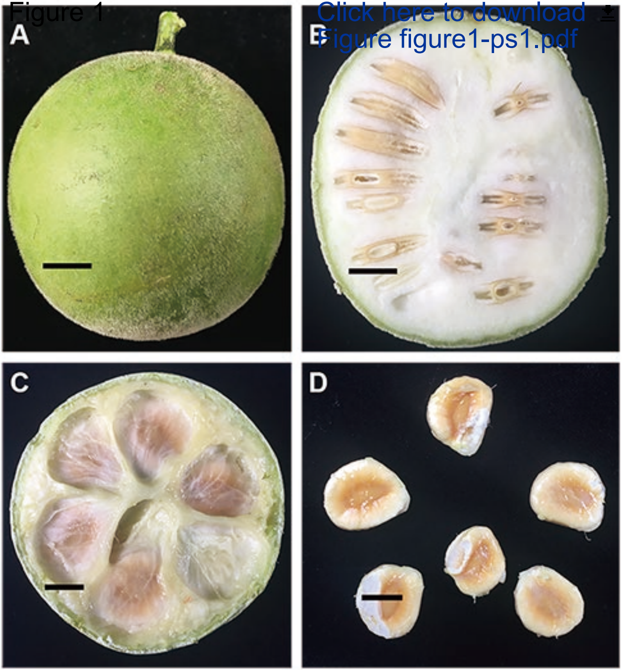

Figure 2

Click here to download Figure figure1.pdf

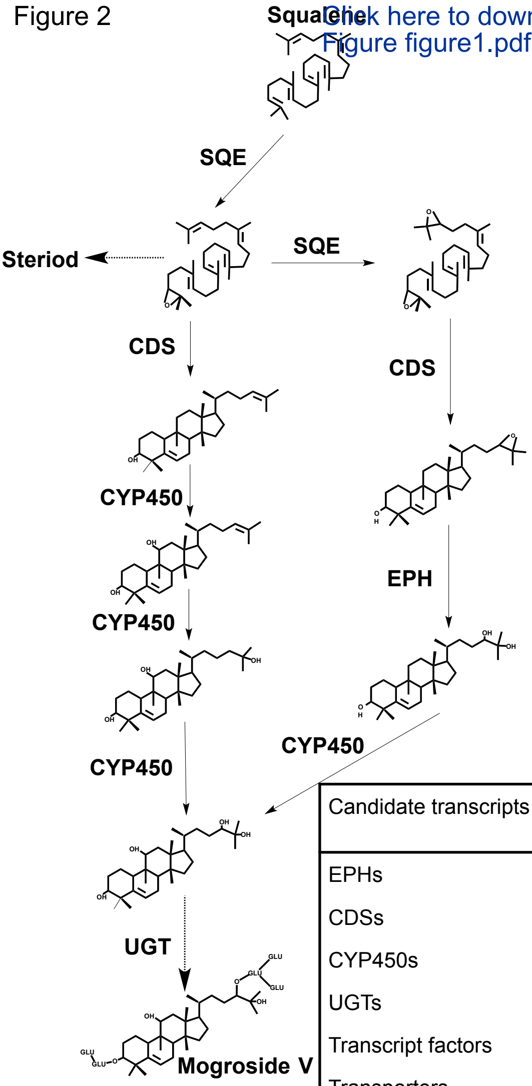

Candidate transcripts	Number
EPHs	5
CDSs	6
CYP450s	19
UGTs	6
Transcript factors	64
Transporters	72
Other enzymes	311

Figure 3

Click here to download Figure figure2.pdf

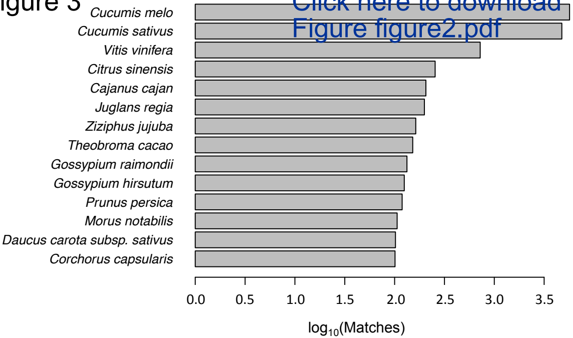

Figure 4

Click here to download Figure figure3.pdf

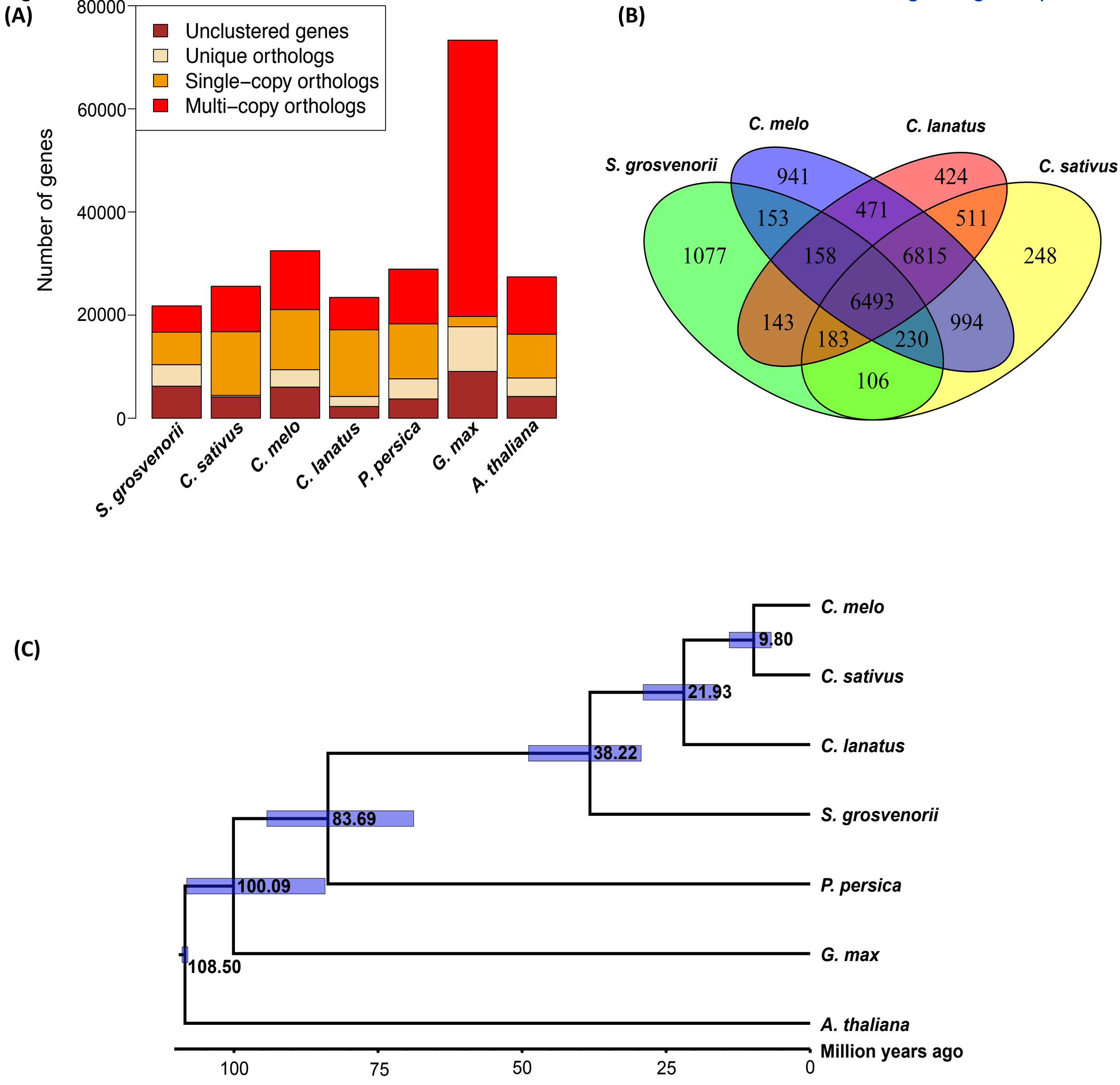

Figure 5

Click here to download Figure figure4.pdf

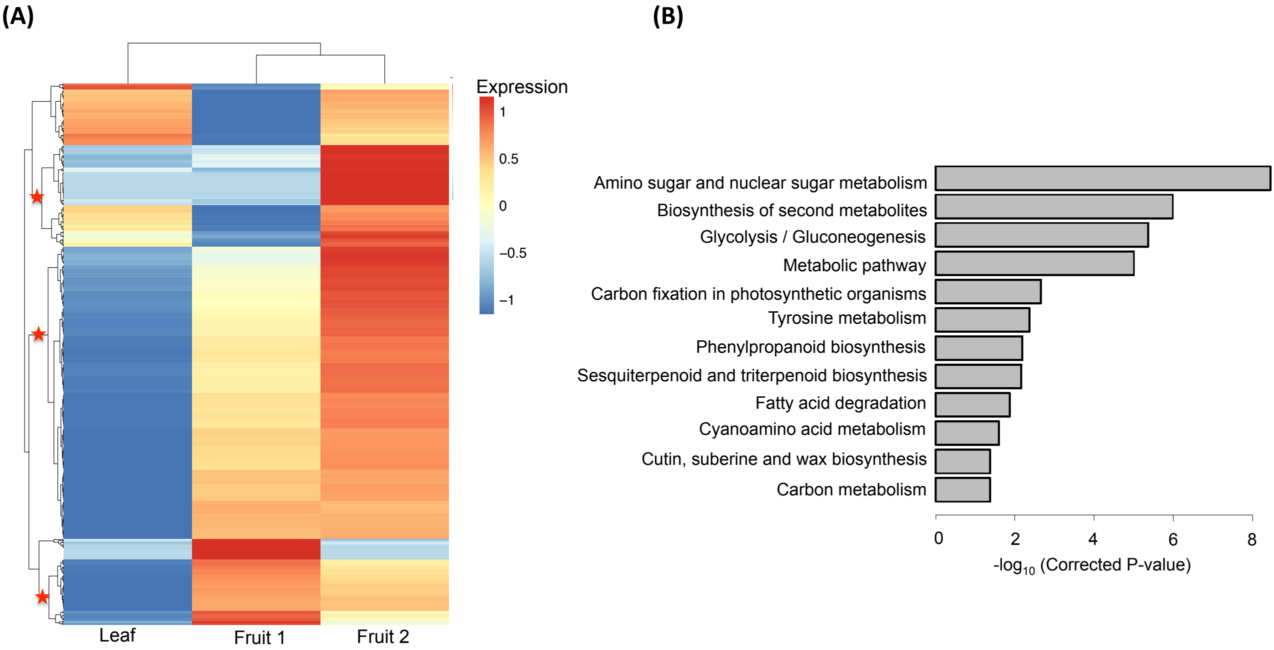

Supplement: GIGA-D-17-00311_Original_Submission.pdf [file giy067_giga-d-17-00311_original_submission.pdf]
