# Supplementary material for: Improved de novo genome assembly and analysis of the Chinese cucurbit Siraitia grosvenorii, also known as monk fruit or luo-han-guo
Source: Gigascience. 2018 Jun 8;7(6):giy067. doi: 10.1093/gigascience/giy067 (PMC6007378; doi:10.1093/gigascience/giy067)

GigaScience

Improved de novo genome assembly and analysis of the Chinese cucurbit Siraitia grosvenorii, also known as monk fruit or luo-han-guo

--Manuscript Draft--

Manuscript Number:	GIGA-D-17-00311R2
Full Title:	Improved de novo genome assembly and analysis of the Chinese cucurbit Siraitia grosvenorii, also known as monk fruit or luo-han-guo
Article Type:	Data Note
Funding Information:	National Key R&D Program of China (2017YFA0503800) — Pro. Xing Wang Deng
Abstract:	Background: Luo-han-guo (Siraitia grosvenorii), also called monk fruit, is a member of the Cucurbitaceae family. Currently, monk fruit has become important for research because of the pharmacological and economic potential of its non-caloric, extremely sweet components (mogrosides). It is also commonly used in traditional Chinese medicine for the treatment of lung congestion, sore throat and constipation. Recently, a single reference genome became available for monk fruit, assembled from 36.9 x genome coverage reads via Illumina sequencing platforms. This genome assembly has a relatively short (34.2 Kb) contig N50 length and lacks integrated annotations. These drawbacks make it difficult to use as a reference in assembling transcriptomes and discovering novel functional genes.<br>Findings: Here, we offer a new high-quality draft of the S. grosvenorii genome assembled using 31 Gb (~ 73.8 x) long single molecule real time sequencing (SMRT) reads and polished with ~ 50 Gb Illumina paired-end reads. The final genome assembly is approximately 469.5 Mb, with a contig N50 length of 432,384 bp, representing a 12.6-fold improvement. We further annotated 237.3 Mb of repetitive sequence and 30,565 consensus protein coding genes with combined evidence. Phylogenetic analysis showed that S. grosvenorii diverged from members of the Cucurbitaceae family approximately 40.9 million years ago. With comprehensive transcriptomic analysis and differential expression testing, we identified 4,606 up-regulated genes in the early fruit compared to the leaf, a number of which were linked to metabolic pathways regulating fruit development and ripening.<br>Conclusions: The availability of this new monk fruit genome assembly, as well as the annotations, will facilitate the discovery of new functional genes and the genetic improvement of monk fruit.<br>Keywords: Siraitia grosvenorii, Monk fruit, PacBio sequencing, Ortholog analysis, RNA-Seq, Mogrosides biosynthesis
Corresponding Author:	Hang He<br><br>beijing, Beijing CHINA
Corresponding Author Secondary Information:	
Corresponding Author's Institution:	
Corresponding Author's Secondary Institution:	
First Author:	Mian Xia
First Author Secondary Information:	
Order of Authors:	Mian Xia<br>Xue Han<br>Hang He<br>Renbo Yu<br>Gang Zhen<br>Xiping Jia<br>Beijiu Cheng

Powered by Editorial Manager® and ProduXion Manager® from Aries Systems Corporation

	Xing Wang Deng
**Order of Authors Secondary Information:**	
**Response to Reviewers:**	Hans Zauner<br>Assistant Editor<br>GigaScience<br><br>Dear Dr. Zauner,<br><br>Thank you for handing out our revised manuscript entitled "Improved de novo genome assembly and analysis of the Chinese cucurbit Siraitia grosvenorii, also known as monk fruit or luo-han-guo" (GIGA-D-17-00311). We have removed the highlights of changes as your request and updated the files the data curator suggested in the e-mail at the first time he contacted us. We also added the citation of GigaDB in this revised manuscript, but we still do not know the doi link. In addition, we thank the reviewer 2 for the comment of assembling long contigs using paired-end reads to measure the consensus quality of our final assembly. We did not use this method to assess the genome quality because we have measured the sequence accuracy using paired-end reads mapping to the assembly, and the second method was widely used in genome quality assessment.
**Additional Information:**	
**Question**	**Response**
Are you submitting this manuscript to a special series or article collection?	No
**Experimental design and statistics**<br><br>Full details of the experimental design and statistical methods used should be given in the Methods section, as detailed in our Minimum Standards Reporting Checklist. Information essential to interpreting the data presented should be made available in the figure legends.<br><br>Have you included all the information requested in your manuscript?	Yes
**Resources**<br><br>A description of all resources used, including antibodies, cell lines, animals and software tools, with enough information to allow them to be uniquely identified, should be included in the Methods section. Authors are strongly encouraged to cite Research Resource Identifiers (RRIDs) for antibodies, model organisms and tools, where possible.<br><br>Have you included the information requested as detailed in our Minimum Standards Reporting Checklist?	Yes
**Availability of data and materials**	Yes

All datasets and code on which the conclusions of the paper rely must be either included in your submission or deposited in publicly available repositories (where available and ethically appropriate), referencing such data using a unique identifier in the references and in the "Availability of Data and Materials" section of your manuscript.<br><br>Have you have met the above requirement as detailed in our Minimum Standards Reporting Checklist?	

Powered by Editorial Manager® and ProduXion Manager® from Aries Systems Corporation

Manuscript

Click here to download Manuscript Second_revied_giga_manuscript.docx

Improved *de novo* genome assembly and analysis of the Chinese cucurbit *Siraitia grosvenorii*, also known as monk fruit or luo-han-guo

Mian Xia[1,†], Xue Han[2,†], Hang He[2,†], Renbo Yu[2], Gang Zhen[2], Xiping Jia[3], Beijiu Cheng[1,*] and Xing Wang Deng[2,*]

[1]Key Laboratory of Crop biology of Anhui Province, Anhui Agricultural University, Hefei, China

[2]School of Advanced Agriculture Sciences and School of Life Sciences, State Key Laboratory of Protein and Plant Gene Research, Peking University, Beijing 100871, China

[3]National Demonstration Area of Modern Agriculture in Cangxi, Sichuan Province, China

[*]Correspondence: Xing Wang Deng (deng@pku.edu.cn), Beijiu Cheng (cbj@ahau.edu.cn)

[†]Theses authors contributed equally to this article.

ORCIDs: Hang He: 0000-0003-3165-283X; Beijiu Cheng: 0000-0003-4518-2381.

Abstract

Background: Luo-han-guo (*Siraitia grosvenorii*), also called monk fruit, is a member of the Cucurbitaceae family. Monk fruit has become an important area for research because of the pharmacological and economic potential of its non-caloric, extremely sweet components (mogrosides). It is also commonly used in traditional Chinese medicine for the treatment of lung congestion, sore throat and constipation. Recently, a single reference genome became available for monk fruit, assembled from 36.9 x genome coverage reads via Illumina sequencing platforms. This genome assembly has a relatively short (34.2 Kb) contig N50 length and lacks integrated

annotations. These drawbacks make it difficult to use as a reference in assembling transcriptomes and discovering novel functional genes.

Findings: Here, we offer a new high-quality draft of the *S. grosvenorii* genome assembled using 31 Gb (~ 73.8 x) long single molecule real time sequencing (SMRT) reads and polished with ~ 50 Gb Illumina paired-end reads. The final genome assembly is approximately 469.5 Mb, with a contig N50 length of 432,384 bp, representing a 12.6-fold improvement. We further annotated 237.3 Mb of repetitive sequence and 30,565 consensus protein coding genes with combined evidence. Phylogenetic analysis showed that *S. grosvenorii* diverged from members of the Cucurbitaceae family approximately 40.9 million years ago. With comprehensive transcriptomic analysis and differential expression testing, we identified 4,606 up-regulated genes in the early fruit compared to the leaf, a number of which were linked to metabolic pathways regulating fruit development and ripening.

Conclusions: The availability of this new monk fruit genome assembly, as well as the annotations, will facilitate the discovery of new functional genes and the genetic improvement of monk fruit.

Keywords: *Siraitia grosvenorii*, Monk fruit, PacBio sequencing, Ortholog analysis, RNA-Seq, Mogrosides biosynthesis

Data description

Introduction

Siraitia grosvenorii (luo-han-guo or monk fruit, NCBI Taxonomy ID: 190515) is an herbaceous perennial native to southern China and is a famous specialty in Guilin city, Guangxi Province of

China (Figure 1)[1]. In addition to being used as a natural sweetener, *S. grosvenorii* has been used in China as a folk remedy for the treatment of lung congestion, sore throat and constipation for hundreds of years [2]. The ripe fruit of *S. grosvenorii* contains mogrosides, which have become a popular research topic due to their pharmacological characteristics, including putative anti-cancer properties [3]. Additionally, mogrosides are purified and used as a non-caloric, non-sugar sweetener in the United States and Japan, as they are estimated to be approximately 300 times as sweet as sucrose [1,4]. To date, *S. grosvenorii* fruit was shown to have additional pharmacological effects and contain different types of secondary metabolites [5,6]. Monk fruit products have been approved as dietary supplements in Japan, the US, New Zealand and Australia [2,7].

The biosynthesis pathway of mogrosides has been extensively studied, and several genes have been identified [8-11]. Squalene is thought to be the initial substrate and precursor for triterpenoid and sterol biosynthesis. Squalene epoxidases (SQE) perform epoxidation, which creates squalene or oxidosqualene, and cucurbitadinenol synthase (CDS) cyclizes oxidosqualene to form the cucurbitadienol triterpenoid skeleton, which is a distinct step in phytosterol biosynthesis [12]. Epoxide hydrolases (EPH) and cytochrome P450s (CYP450) further oxidize cucurbitadienols to produce mogrol, which is glycosylated by UDP-glycosyl-transferases (UGT) to form mogroside V (Figure 2).

The genome of *S. grosvenorii* was first published in 2016 and served the purpose of identifying the genomic organization of the gene families of interest but did not act as the reference in the transcriptome assembly and gene families identification [8]. Although the first draft genome

assembly was a useful resource, some improvements remain necessary, including improving the continuity and completeness, genome assembly assessment, annotation of genes and repetitive regions, and analysis of other genomic features. With an average read length now exceeding 10 Kb, SMRT sequencing technology from Pacific Biosciences (PacBio) has the potential to significantly improve genome assembly quality [13]. Therefore, we *de novo* assembled a high-quality genome draft of *S. grosvenorii* using high-coverage PacBio long reads and applied extensive genomic and transcriptomic analyses. This new assembly, annotations and other genomic features discussed below will serve as valuable resources for investigating the economic and pharmacological characteristics of monk fruit and will also assist in the molecular breeding of monk fruit.

DNA libraries construction and sequencing

A total of 20 μg of genomic DNA was extracted from seedlings of *S. grosvenorii* (variety Qingpiguo) using a modified CTAB method [14] to construct 2 libraries with an insert size of 20 Kb. The plants were introduced from the Yongfu District (Guangxi Province, China) and planted in Cangxi County (Sichuan Province, China). Sequencing of *S. grosvenorii* was performed using the Pacbio RSII platform (Pacific Biosciences; USA) and generated 31 Gb (~ 73.8 x) of data from 44 SMRT cells, with an average subread length of 7.7 Kb and read quality of 82% after filtering out low-quality bases and adapters (Table 1).

A total of 300 ng of genomic DNA was extracted as described above, and the library was constructed using DNA sequence fragments of ~470 bp, with an approximate insert size of 350

bp. Sequencing was performed using a 2x150 paired-end (PE) configuration, and base calling was conducted using the HiSeq Control Software (HCS) + OLB + GAPipeline-1.6 (Illumina; CA, USA) on the HiSeq instrument, which generated a total of 169 M (over 100 x) short reads.

RNA isolation and sequencing

Fresh roots, leaves and early fruit of *S. grosvenorii* were sampled in our garden in Cangxi County. All samples were stored at -80 ºC after immediate treatment with liquid nitrogen. Total RNA was isolated from (1) leaves of female plants (FL), (2) leaves of male plants (ML), (3) leaves beside fruits (L), (4) roots(R), (5) fruit of 3 DAA (F1) and (6) fruit of 20 DAA (F2) using the Qiagen RNeasy Plant Mini Kits (Qiagen; CA, USA). Paired-end libraries (PE150 with an insert size of 350 bp) were constructed and subsequently sequenced via the Illumina HiSeq X-Ten platform (Illumina; CA, USA).

Table 1 SMRT reads used for genome assembly

**Statistics**	**Length (bp)**
Total raw data	31 G
Mean length of raw reads	11 K
N50 of raw reads	15,754
Mean length of subreads	7.7 K
N50 of subreads	11,898

Subreads: reads without adapters and low-quality bases.

Genome assembly

Initial correction of long reads was performed using FALCON (Falcon, RRID:SCR_016089)[15]

with _cutoff length = 5000 according to the distribution of read lengths and -B15, -s400 to cut reads into blocks of 400 Mb and align 15 blocks to another block at the same time. The 25x coverage of the longest corrected reads was extracted with Perl scripts and assembled by mecat2canu command of MECAT [16] with GenomeSize=420000000 estimated in the previous study [8]. This led to a new genome assembly of 467 Mb with a contig N50 size of 434,684 bp (Table 2). This genome size was slightly larger than the estimated 420 Mb [8], which was likely due to the high genome heterozygosity. We used the consensus algorithm Quiver [15] and further polished the assembly with paired-end reads using Pilon (Pilon, RRID:SCR_014731)[17]. The final assembly produced 4,128 contigs, 614 of which were over 100 Kb long, with a contig N50 length of 432,384 bp (Table 2). Compared to the preliminary draft of the published *Siraitia* genome, the contiguity was improved more than ~12.6 times.

Table 2 Metrics of *de novo S. grosvenorii* genome assembly

**Statistics**	**Contig**	**Contig (Polished)**
Total number	4,128	4128
Total length (bp)	467,072,951	469,518,713
N50 length (bp)	433,684	432,384
N90 length (bp)	36,820	36,953
Max length (bp)	7,657,852	7,683,850
GC content (%)	33.57	33.49

Genome assessment

We estimated the completeness of the assembly using Benchmarking Universal Single-Copy Orthologues (BUSCO v2, RRID:SCR_015008) [18] analysis. Of the 1,440 orthologues identified in plants, 1,284 were found in the genome assembly, including 849 in single-copy and 435 in multi-copy (Table 3). In addition, we used RNA-Seq data from different organs to assess the sequence quality. All 15 RNA-Seq libraries were mapped to the assembly using HISAT2 (HISAT2 , RRID:SCR_015530) [19], and the overall alignment rate for each data was used as a rough estimation of sequence quality. We also estimated the base error rate of the assembly with both DNA paired-end reads and published DNA short reads [8]. We used BWA-mem (http://bio-bwa.sourceforge.net/) to align both short reads to the genome assembly and filtered out low-quality (mapping quality < 30) alignments with SAMtools (SAMtools, RRID:SCR_002105) [20]. Then, we used the Genome Analysis Toolkit (GATK, RRID:SCR_001876) HaplotypeCaller [21] to call short variants. The GATK VariantFiltration program was used to filter out low-quality variants with the following expression: QD < 2.0 || ReadPosRankSum < -8.0 || FS > 60.0 || QUAL < 50 || DP < 10. Coverage of each alignment file was scanned using Qualimap 2 [22], and the error rate was calculated as the average number of short variants that appear at both alleles (labeled as 1/1 and 1/2 in Table 5) per base. The overall alignment rates of reads in all samples were over 80% (Table 4), and the average base error rate was estimated as less than 1E-3, which suggests a high-quality assembly (Table 5).

Table 3 Summarized benchmarks of the BUSCO assessment

	Monk fruit (%)
Complete BUSCOs	89.2
Complete and single-copy	59.0
Complete and duplicated	30.2
Partial	2.7
Missing	8.1

Table 4 Quality evaluation of the draft genome with the overall alignment rate

Sample	Overall alignment rate
FL-1	89.93%
FL-2	87.75%
FL-3	85.83%
ML-1	89.70%
ML-2	89.73%
ML-3	85.07%
L-1	85.95%
L-2	87.39%
R-1	81.50%
R-2	84.36%
R-3	84.57%
F1-1	84.35%
F1-2	91.58%
F2-1	86.83%
F2-2	87.37%

FL: female leaf, ML: male leaf, L: leaf, R: root, F1: fruit stage 1, F2: fruit stage 2

Table 5 Genome base accuracy estimated using resequencing short reads

**Sample**	**Mean Depth**	**Coverage**	**Number of Variation**				**Error rate**
			**0/1**	**1/1**	**1/2**	**Total**	
Paired-end	65.3 x	92.99%	1,342,849	37,987	14,704	1,395,540	1.21E-4
Published	80.0 x	90.79%	2,569,592	172,906	16,777	2,759,276	4.45E-4

High-quality genome criteria: 1E-4.

0: genotype that is identical to the reference, 1,2: genotype that is different from the reference.

Error rate = (Number of 1/1 + Number of 1/2) / (Genome size * Coverage).

Repeat annotation

We scanned the genome using RepeatMasker (RepeatMasker, RRID:SCR_012954) [23] with Repbase [24] and a *de novo* repeat database constructed with RepeatModeler (RepeatModeler, RRID:SCR_015027) [25]. Sequences 240 Mb (51.14% of the assembled genome) in length were identified as repetitive elements, which was slightly larger than the 42.8% of *Momordica charantia* [26] and much larger than the 28.2% of *Cucumis sativus* [27]. We further classified the repetitive regions and found that the vast majority were interspersed repeats. Among them, the main subtypes were unclassified repeats and long terminal repeats (LTRs), with Copia (27.1 Mb, 5.8% of the genome) and Gypsy (38.6 Mb, 8.2% of the genome) LTRs being the most abundant. Compared to cucumber, the genome enlargement in monk fruit and bitter gourd was likely driven by the expansion of interspersed repeats (Table 6).

Gene annotation

To generate gene models, the *S. grosvenorii* genome was annotated using 3 gene prediction

pipelines including homology-based, *de novo* and RNA-Seq data-based prediction. First, we aligned the 3 cucurbitaceous proteomes downloaded from the cucurbit database (http://cucurbitgenomics.org, cucumber_Chinese_Long_v2, melon_v3, and watermelon_97103_v1) to the genome assembly using TBLASTN with an E-value of 1e-5 and filtering out bad hits (identity < 50% and length < 50%). The best hit of each retained protein was extracted and further used to predict protein coding gene structures with GeneWise (GeneWise, RRID:SCR_015054, https://www.ebi.ac.uk/~birney/wise2/) [28]. Second, we *de novo* predicted protein coding genes using AUGUSTUS (AUGUSTUS , RRID:SCR_008417) [29] with the repeat masked genome. Third, we used StringTie [30] to assemble 15 RNA-Seq alignment files (described above) generated from HISAT2 using the assembly as the reference, and TransDecoder (https://github.com/TransDecoder/TransDecoder) to generate an annotation file based on transcripts. Finally, the three respective annotation files were combined using EVidenceModeler (EVM, RRID:SCR_014659) [31]. After combining these gene structure predictions, we obtained 30,565 consensus protein-coding genes (Table 7). We annotated the genes using BLASTp searching against the NCBI non-redundant protein database (nr) and found that 78.3% of the predicted genes had at least one significant homologue (E-value less than 1E-3), indicating that the gene structures were credible. We found that the majority of homologous proteins belonged to cucurbitaceous plants, such as cucumber and muskmelon (Figure 3). Protein domain and gene ontology (GO) term annotations were performed using InterProScan 5 (InterProScan, RRID:SCR_005829, Table 7) [32]. In addition, genes annotated as SQEs, EPHs, CDSs, EPHs, CYP450s, and UGTs were compared with those in other Cucurbitaceae genomes, and we found

that gene abundance in the 5 mogroside-related gene families were not significantly different among *S. grosvenorii*, *Cucumis sativus*, *Cucurbita moschata* and *Cucurbita maxima* (http://cucurbitgenomics.org, Table 8).

Table 6 Repeat annotation of the *S. grosvenorii* genome

Repeat Classification		***S. grosvenorii***		***M. charantia***		***C. sativus***	
		Length (bp)	Content	Length (bp)	Content	Length (bp)	Content
Interspersed repeats	SINEs	0	0.00%	0	0.00%	0	0.00%
	LINEs	9,629,949	2.05%	5,183,926	1.82%	2,397,830	1.22%
	LTR	67,499,840	14.38%	34,217,647	11.98%	8,253,090	4.18%
	DNA elements	9,372,444	2.00%	3,460,431	1.21%	2,777,943	1.41%
	Unclassified	147,311,542	31.38%	75,056,338	26.28%	37,539,553	19.03%
	Total	233,813,775	49.80%	117,918,342	41.29%	50,967,966	25.84%
Simple repeats		5,401,880	1.15%	3,451,508	1.21%	3,547,474	1.80%
Low complexity		1,570,875	0.33%	958,289	0.34%	1,095,406	0.56%
Total		240,122,745	51.14%	122,111,538	42.75%	55,540,243	28.15%

Ortholog analysis

Gene family clustering analysis was accomplished using OrthoMCL (OrthoMCL, RRID:SCR_007839) [33] on protein sequences of *S. grosvenorii*, *C. sativus* (cucumber_ChineseLong_v2, http://cucurbitgenomics.org/) [27], *Cucumis melo* (CM3.5.1, http://cucurbitgenomics.org/) [34], *Citrullus lanatus* (watermelon_97103_v1, http://cucurbitgenomics.org/) [35], *Prunus persica* (Prunus_persica.prupe1_0, https://plants.ensembl.org/) [36], *Solanum lycopersicum* (Solanum_lycopersicum.SL2.50,

http://plants.ensembl.org/) [37], *Arabidopsis thaliana* (Tair10, http://Arabidopsis.org/) [38] and *Oryza sativa* (Oryza_sativa.IRGSP-1.0, https://plants.ensembl.org/) [39]. A total of 23,246 *S. grosvenorii* genes were clustered into 26,190 gene families, including 1,471 unique *S. grosvenorii* gene families (Figure 4A). Compared to other cucurbitaceous plants, *S. grosvenorii* shares fewer gene families with relative species (Figure 4B), indicating an earlier divergence time than *C. lanatus*. A total of 834 single-copy gene families were identified and selected to construct the phylogenetic tree using RAxML (RAxML, RRID:SCR_006086) [40]. We used Muscle (Muscle, RRID:SCR_011812, https://www.ebi.ac.uk/Tools/msa/muscle/) [41] to align the orthologs, and the alignment was treated with Gblocks [42] with parameters of -t=p -b5=h -b4=5 –b3=15 -d=y -n=y. The divergence time was estimated by MCMCtree [43]. Phylogenetic analysis showed that *S. grosvenorii* diverged from the Cucurbitaceae family approximately 40.95 million years ago (Figure 4C).

Table 7 Gene prediction and annotation

	**RNA-Seq data-based**	**Ab initio**	**Homology-based**	**Integration**	**Annotation**		
**Weight**	10	0.1	5	-	-		
					nr	IPR	GO
**Number of predicted genes**	27,304	60,818	130,686	30,565	23,936	19,684	14,966
**Tools**	HISAT2<br>StringTie<br>TransDecoder	RepeatMasker<br>AUGUSTUS	BLAST<br>GeneWise	EVM	BLAST	InterProScan	

Table 8 Abundance analysis of the mogrosides synthesis related gene families

	S. grosvenorii	*C. sativus*	*C. moschata*	*C. maxima*
SQE	5 (5)	1	2	1
EPH	30 (8)	23	29	22
CYP450	276 (191)	213	289	234
UGT	156 (131)	124	137	121
CDS	1 (1)	1	2	3

The numbers quoted are the number of genes belonging to each gene family annotated in monk fruit genome version 1.

Transcriptomic analysis

Mogrosides are produced during fruit development in *S. grosvenorii* and are not found in vegetative tissues [8]. Thus, we performed an extensive transcriptomic analysis of early fruit at 2 stages (stage 1 sampled at 3 days after anthesis and stage 2 sampled at 20 days after anthesis) and of leaves to identify transcripts involved in mogrosides synthesis in early fruit. Using the genome-wide annotation, RNA-Seq reads were mapped to the genome assembly, and read count tables were generated using HISAT2 and StringTie [30] for the next step of differential expression analysis. DESeq2 (RRID:SCR_000154) [44] was used to detect differential gene expression among leaves (L), fruit of 3 DAA (F1) and fruit of 20 DDA (F2) with the criteria of padj < 0.01 and |log2FoldChange| > 1. Genes that were up-regulated with fruit development were merged and used for KEGG pathway enrichment analysis with KOBAS (KOBAS, RRID:SCR_006350) [45]. Thirteen pathways were significantly enriched (Corrected P-value < 0.01), and the most enriched

pathways were related to metabolic pathways. In particular, the sesquiterpenoid and triterpenoid biosynthesis pathways were significantly enriched, indicating that genes involved in the biosynthesis of secondary metabolites, including mogrosides, perform their functions in the very early fruit (Figure 5). Genes possibly related to mogrosides biosynthesis in early fruit according to the gene annotation were assigned to the mogrosides synthesis pathway (Figure 2).

Discussion

Siraitia grosvenorii is an important herbal crop with multiple economic and pharmacological values. Mogrosides, the main effective components of *S. grosvenorii* fruit, are partial substitutes of sucrose because of its extremely sweet and non-caloric characteristics as more progress is made on molecular breeding and purification processes. Additionally, monk fruit could serve in contrast to other cucurbitaceous plant because of its earlier divergence from the common ancestor than some other well-studied cucurbits (cucumber, muskmelon), and it may be a new system for the investigation of plant sex determination. In the present study, we sequenced and assembled the second version of the monk fruit genome. With a great improvement in completeness and accuracy, the genome as well as the annotations will provide valuable resources and reference information for transcriptome assembly and novel gene discovery. These resources and further transcriptomics analysis of ripe fruit and young fruit will facilitate studies of the secondary metabolite synthesis pathways and monk fruit breeding.

Availability of supporting data

The genomic and transcriptomic sequencing reads were deposited in the Genome Sequence Archive (GSA) under the Accession number CRA000522 and ENA (European Nucleotide Archive) under the Accession number PRJEB23465, PRJEB23466, PRJEB25737. Supporting data are also available in the *GigaScience* database, GigaDB [46].

ACKNOWLEDGMENTS

This research was supported by the National Key R&D Program of China (2017YFA0503800) to X.W.D. and in part by the National Demonstration Area of Modern Agriculture in Cangxi, Sichuan Province, China.

Author's contribution

XWD, BC, HH, and MX planned and coordinated the project. MX collected and grew the plant material. RY and GZ collected the samples and performed experiments. Genome assembly, annotation, phylogenetic analysis and manuscript writing were completed by XH, MX, HH and XWD.

Competing interests

The authors declare that they have no competing interests.

Reference

1. Zhang JS, Dai LH, Yang JG, Liu C, Men Y, Zeng Y, et al. Oxidation of cucurbitadienol catalyzed by

CYP87D18 in the biosynthesis of mogrosides from *Siraitia grosvenorii.* Plant Cell Physiol. 2016;57:1000-1007.

2. Li C, Lin LM, Sui F, Wang ZM, Huo HR, Dai L, et al. Chemistry and pharmacology of Siraitia grosvenorii: A review. Chinese Journal of Natural Medicines. 2014;12:89-102.
3. Liu C, Dai LH, Dou DQ, Ma LQ, Sun YX. A natural food sweetener with anti-pancreatic cancer properties. Oncogenesis. 2016;5:e217.
4. Nie RL. The decadal progress of triterpene saponins from *Cucurbitaceae* (1980–1992). Acta Bot Yunnan 1994;16:201–208.
5. Wang Q, Qin HH, Wang W, Qiu SP. The pharmacological research progress of *Siraitia grosvenorii.* J Guangxi Tradit Chin Med Univ. 2010;13:75-76.
6. Zhang H, Li XH. Research progress on chemical compositions of Fructus Momordicae. J Anhui Agri Sci. 2011;39:4555-4556, 4559.
7. Pawar RS, Krynitsky AJ, Rader JI. Sweeteners from plants--with emphasis on Stevia rebaudiana (Bertoni) and Siraitia grosvenorii (Swingle). Anal Bioanal Chem. 2013 ;405:4397-407.
8. Itkin M, Davidovich-Rikanati R, Cohen S, Portnoy V, Doron-Faigenboim A, Oren E, et al. The biosynthetic pathway of the nonsugar, high-intensity sweetener mogroside V from *Siraitia grosvenorii*. Proc Natl Acad Sci U S A. 2016;113:E7619-E7628.
9. Dai LH, Liu C, Zhu YM, Zhang JS, Men Y, Zeng Y, et al. Functional characterization of cucurbitadienol synthase and triterpene glycosyltransferase involved iin biosynthesis of mogrosides from *Siraitia grosvenorii*. Plant Cell Physiol. 2015;56: 1172-1182.

10. Zhang JS, Dai LH, Yang JG, Liu C, Men Y, Zeng Y, et al. Oxidation of cucurbitadienol catalyzed by CYP87D18 in the biosynthesis of mogrosides from *Siraitia grosvenorii.* Plant Cell Physiol. 2016;57:1000-1007.

11. Tang Q, Ma XJ, Mo CM, Wilson WI, Song C, Zhao H, et al. An efficient approach to finding *Siraitia grosvenorii* triterpene biosynthetic genes by RNA-seq and digital gene expression analysis. BMC Genomics. 2011;12: 343.

12. Shibuya M, Adachi S, Ebizuka Y. Cucurbitadienol synthase, the first committed enzyme for cucurbitacin biosynthesis, is a distinct enzyme from cycloartenol synthase for phytosterol biosynthesis. Tetrahedron. 2004;60: 6995–7003.

13. Zimin AV, Stevens KA, Crepeau MW, Puiu D, Wegrzyn JL, Yorke JA, et al. An improved assembly of the loblolly pine mega-genome using long-read single-molecule sequencing. Gigascience. 2017;6:1-4.

14. Porebski S, Bailey LG, Baum BR. Modification of a CTAB DNA extraction protocol for plants containing high polysaccharide and polyphenol components. Plant Mol Biol Rep. 1997;15:8-15.

15. Chin CS, Alexander DH, Marks P, Klammer AA, Drake J, Heniner C, et al. Nonhybrid, finished microbial genome assemblies from long-read SMRT sequencing data. Nat Methods. 2013;10:563-9.

16. Xiao CL, Chen Y, Xie SQ, Chen KN, Wang Y, Han Y, et al. MECAT: fast mapping, error correction, and de novo assembly for single-molecule sequencing reads. Nat Methods. 2017;14:1072-1074.

17. Walker BJ, Abeel T, Shea T, Priest M, Abouelliel A, Sakthikumar S, et al. Pilon: an integrated tool for comprehensive microbial variant detection and genome assembly improvement. PLoS One. 2014;9:e112963.

18. Simão FA, Waterhouse RM, Ioannidis P, Kriventseva EV, Zdobnov EM. BUSCO: assessing genome assembly and annotation completeness with single-copy orthologs. Bioinformatics. 2015;31:3210-2.

19. Kim D, Langmead B, Salzberg SL. HISAT: a fast spliced aligner with low memory requirements. Nat Methods. 2015;12:357-60.

20. Li H, Handsaker B, Wysoker A, Fennell T, Ruan J, Homer N, et al. The Sequence alignment/map (SAM) format and SAMtools. Bioinformatics. 2009;25:2078-9.

21. McKenna A, Hanna M, Banks E, Sivachenko A, Cibulskis K, Kernytsky A, et al. The Genome Analysis Toolkit: a MapReduce framework for analyzing next-generation DNA sequencing data. Genome Res. 2010;20:1297-303.

22. Okonechnikov K, Conesa A, García-Alcalde F. Qualimap 2: advanced multi-sample quality control for high-throughput sequencing data. Bioinformatics. 2016;32:292-4.

23. Tarailo-Graovac M, Chen N. Using RepeatMasker to identify repetitive elements in genomic sequences. Curr Protoc Bioinformatics. 2009;3:4–14.

24. Visser M, Van der Walt AP, Maree HJ, Rees DJ G, Burger JT. Extending the sRNAome of apple by next-generation sequencing. PLoS one. 2014;9:e95782.

25. Smit A, Hubley R. RepeatModeler Open-1.0.8, 2008; http://www.repeatmasker.org/RepeatModeler.html.

26. Urasaki N, Takagi H, Natsume S, Uemura A, Taniai, N, Miyagi N, et al. Draft genome sequence of bitter gourd (*Momordica charantia*), a vegetable and medicinal plant in tropical and subtropical regions. DNA Res. 2016;24:51-58.

27. Huang S, Li R, Zhang Z, Li L, Gu X, Fan W, et al. The genome of the cucumber, *Cucumis sativus* L. Nat Genet. 2009;41:1275-81.

28. Gupta V, Estrada AD, Blakley I, Reid R, Patel K, Meyer MD, et al. RNA-Seq analysis and annotation of a draft blueberry genome assembly identifies candidate genes involved in fruit ripening, biosynthesis of bioactive compounds, and stage-specific alternative splicing. Gigascience. 2015; 4:5.

29. Stanke M, Tzvetkova A, Morgenstern B. AUGUSTUS at EGASP: using EST, protein and genomic alignments for improved gene prediction in the human genome. Genome Biol. 2006;7:S11.1-8.

30. Pertea M, Pertea GM, Antonescu CM, Chang TC, Mendell JT, Salzberg SL. StringTie enables improved reconstruction of a transcriptome from RNA-seq reads. Nat Biotechnol. 2015;33:290-5.

31. Haas BJ, Salzberg SL, Zhu W, Pertea M, Allen JE, Orvis J, et al. Automated eukaryotic gene structure annotation using EVidenceModeler and the Program to Assemble Spliced Alignments. Genome Biol. 2008;9:R7.

32. Quevillon E, Silventoinen V, Pillai S, Harte N, Mulder N, Apweiler R, et al. InterProScan: protein domains identifier. Nucleic Acids Res. 2005;33:W116-20.

33. Li L, Stoeckert CJJr, Roos DS. OrthoMCL: identification of ortholog groups for eukaryotic genomes. Genome Res. 2003;13:2178-89.

34. Garcia-Mas J, Benjak A, Sanseverino W, Bourgeois M, Mir G, González VM, et al. The genome of melon (*Cucumis melo* L.). Proc Natl Acad Sci U S A. 2012;109:11872-7.

35. Guo S, Zhang J, Sun H, Salse J, Lucas WJ, Zhang H, et al. The draft genome of watermelon (*Citrullus lanatus*) and resequencing of 20 diverse accessions. Nat Genet. 2013;45:51-8.

36. International Peach Genome Initiative, Verde I, Abbott AG, Scalabrin S, Jung S, Shu S, et al. The high-quality draft genome of peach (Prunus persica) identifies unique patterns of genetic diversity, domestication and genome evolution. Nat Genet. 2013;45:487-94.

37. Tomato Genome Consortium. The tomato genome sequence provides insights into fleshy fruit evolution. Nature. 2012;485:635-41.

38. Lamesch P, Berardini TZ, Li D, Swarbreck D, Wilks C, Sasidharan R, et al. The Arabidopsis Information Resource (TAIR): improved gene annotation and new tools. Nucleic Acids Res. 2012;40:D1202-10.

39. International Rice Genome Sequencing Project. The map-based sequence of the rice genome. Nature. 2005;436:793-800.

40. Stamatakis A. RAxML version 8: a tool for phylogenetic analysis and post-analysis of large phylogenies. Bioinformatics. 2014;30:1312-3.

41. Edgar RC. MUSCLE: multiple sequence alignment with high accuracy and high throughput. Nucleic Acids Res. 2004;32:1792-7.

42. Talavera G, Castresana J. Improvement of phylogenies after removing divergent and ambiguously aligned blocks from protein sequence alignments. Syst Biol. 2007;56:564-77.

43. Battististuzzi FU, Billing-Ross P, Paliwal A, Kumar S. Fast and slow implementations of relaxed-clock methods show similar patterns of accuracy in estimating divergence times. Mol Biol Evol. 2011;28:2439-42.

44. Love MI, Huber W, Anders S. Moderated estimation of fold change and dispersion for RNA-seq data with DESeq2. Genome Biol. 2014;15:550.

45. Xie C, Mao X, Huang J, Ding Y, Wu J, Dong S, et al. KOBAS 2.0: a web server for annotation and identification of enriched pathways and diseases. Nucleic Acids Res. 2011;39:W316-22.

46. Xia, M; Han, X; He, H; Yu, R; Zhen, G; Jia, X; Cheng, B; Deng, X, W (2018): Supporting data for "Improved de novo genome assembly and analysis of the Chinese cucurbit Siraitia grosvenorii, also known as monk fruit or luo-han-guo" GigaScience Database. http://dx.doi.org/10.5524/100452

Figure legends

Figure 1 Morphological characteristics of the fruit of *S. grosvenorii (A),* vertical section of fruit of *S. grosvenorii* (B), horizontal section of fruit of *S. grosvenorii* (C) and seeds (D). Size bar, 1 cm.

Figure 2 Candidate genes involved in the mogrosides biosynthesis pathway. Candidate functional genes were annotated as SQEs, EPHs, CDSs, CYP450s and UGTs and assigned to the pathway.

Figure 3 Number of best-matching proteins for each predicted *S. grosvenorii* gene by species.

Figure 4 Comparative genome analysis of the *S. grosvenorii* genome. (A) Orthologue clustering analysis of the protein-coding genes in the *S. grosvenorii* genome. (B) Venn diagram showing shared and unique gene families among four cucurbit plant species. Numbers represent the

number of gene families in unique or shared regions. (C) Phylogenetic tree and divergence time of *S. grosvenorii* and 7 other plant species. The phylogenetic tree was generated from 834 single-copy orthologues using the maximum-likelihood method. The divergence time range is shown in blue blocks. The numbers beside the branching nodes are the predicted divergence time.

Figure 5 KEGG pathway enrichment analysis of candidate functional genes.

Figure 1

Click here to download Figure figure1-ps1.pdf

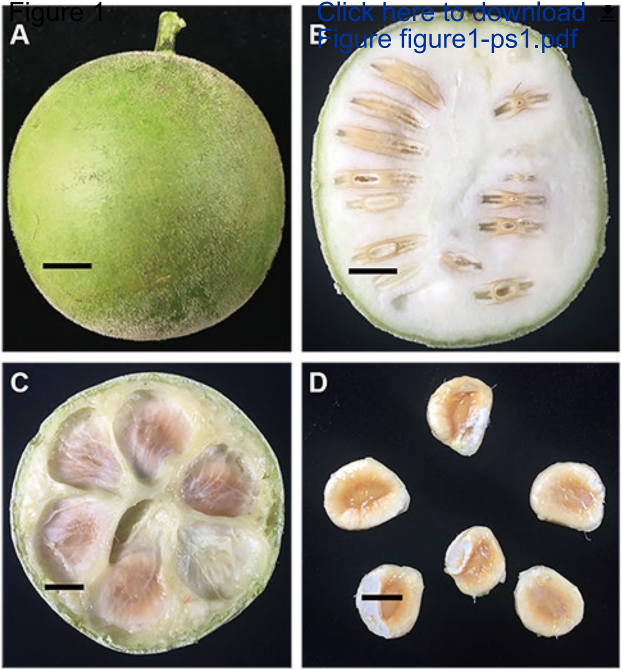

Figure 2

Click here to download Figure

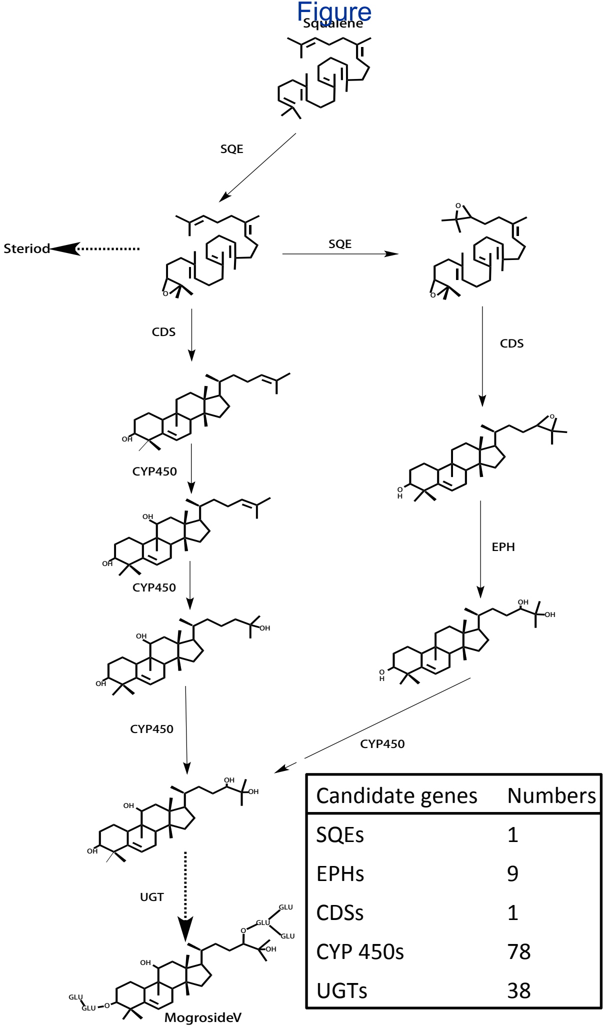

Candidate genes	Numbers
SQEs	1
EPHs	9
CDSs	1
CYP 450s	78
UGTs	38

Figure 3

Click here to download Figure

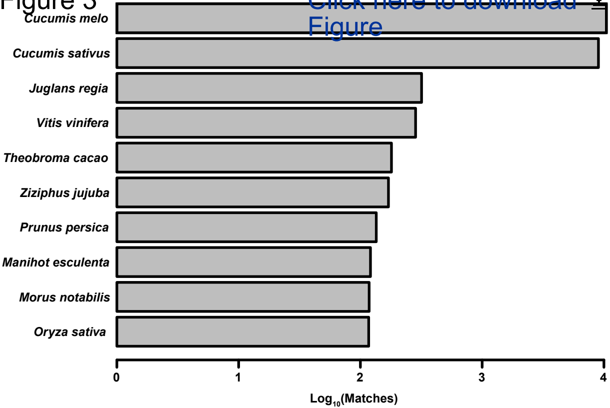

Figure 4

Click here to download Figure Figure4_revised.pdf

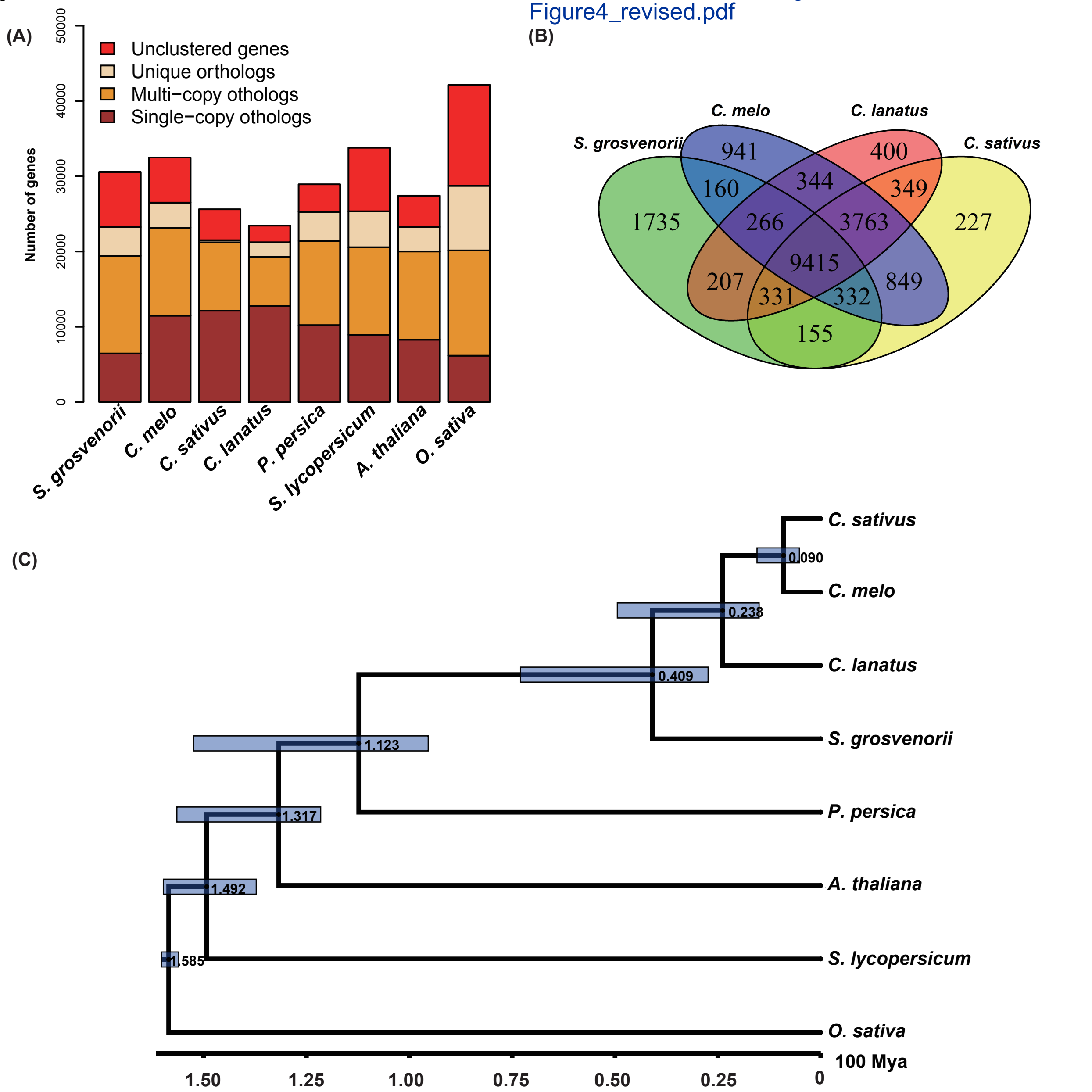

Figure 5

Click here to download Figure

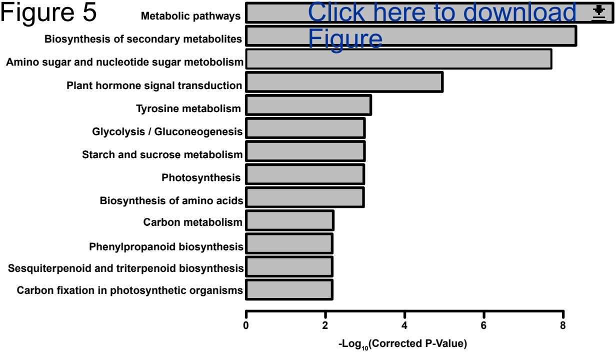

Supplement: GIGA-D-17-00311_Revision_2.pdf [file giy067_giga-d-17-00311_revision_2.pdf]
