# Supplementary material for: Improved de novo genome assembly and analysis of the Chinese cucurbit Siraitia grosvenorii, also known as monk fruit or luo-han-guo
Source: Gigascience. 2018 Jun 8;7(6):giy067. doi: 10.1093/gigascience/giy067 (PMC6007378; doi:10.1093/gigascience/giy067)
Supplement: Response_to_Reviewer_Comments_Original_Submission.pdf [file giy067_response_to_reviewer_comments_original_submission.pdf]

Author's Response To Reviewer Comments

Close

Hans Zauner
Assistant Editor
GigaScience

Dear Dr. Zauner,

Thank you for handing out our manuscript entitled "Improved de novo genome assembly and analysis of the Chinese cucurbit Siraitia grosvenorii, also known as monk fruit or luo-han-guo" (GIGA-D-17-00311). We have revised the manuscript following the suggestions given by the reviewers and the editors.
We carried out assembly polishing with Quiver to correct sequencing errors by aligning PacBio RSII H5 files to the genome sequences and further polishing the assembly using over 100x whole genome Illunima short reads as you suggested. We applied k-mer analysis using whole genome DNA short reads of Qingpiguo to substantiate the high heterozygosity of monk fruit genomes. We also removed some sentences about potential medical benefits of monk fruit in the introduction. All the language problems referred in minor comments have been proofreaded, as well as some confusing sentences. But we were not able to compare the assembly with monk fruit genome version 1 because the first assembly was not publicly available and the authors have not reply to our strong request to their assembly till now. Thus, to assess the quality of out assembly,we calculated base error rate using both our resequencing short reads and their released resequencing data. And the coverage of both dataset were more than 90% of the genome assembly. We also found English native speakers for language editing. We have provided a detailed point-by-point response below and highlighted the changes in red in the revised manuscript.
Reviewer #1 (Major comments):

98:"This genome size was slightly larger than the estimated 420 Mb [8], which was probably due to the high genome heterozygosity." - A k-mer analysis or SNP density analysis should be done and included in the manuscript to substantiate this assertion.

Yes. We have over 100x additional resequencing reads used for k-mer analysis with KmerGenie. The sampled histogram and fit for best k value showed the heterozygous peak substantiate that assertion. In addition, the high genome heterozygosity of monk fruit is observed as it is diecious.

99: Was the genome assembly polished after assembly to correct sequencing errors? This is normally done for PacBio assemblies and should be included in the methods if it was done.
Yes. We performed the assembly using Quiver with raw PacBio RSII H5 files, and polished the assembly using over 100x whole genome Illunima short reads. The polished assembly and annotations have been uploaded to GigaDB.

105/Table 3:13.9% missing BUSCOs seems high for a high coverage PacBio assembly. How does this compare to the original assembly by Itkin et al.?
We analyzed the genome completeness after genome polishing described above, and the missing BUSCOs declined to 8.1%.
We were not able to compare the assembly to the original assembly by Itkin et al., because we cannot obtain the assembly. We sent e-mails to the corresponding author and also PNAS editorial for the

assembly but the authors did not provide it.
In order to compare our assembly with the original assembly by Itkin et al, we aligned both our resequencing short reads and their released whole genome short reads to our assembly using BWA mem program and estimated the average base error rates. They were all less than 1E-3 when using the two datasets as the Table 5 showed in the manuscript, which suggested a high-quality assembly. The differences of base error rates between our resequencing data and the one released earlier were probably due to the variety difference.

Reviewer #1 (Minor momments):

We thank the reviewer for the suggestions on English language, and we have corrected these tissues as suggested one by one and sent the revised manuscript to English native speakers for language editing.

20: platforms
“Platfroms” has been revised as “platforms”.

63: is a useful resource
“Useful resources” has been revised as “a useful resource”.

Table 1: fix units in the table, they are correct in the text
We have checked the units in Table 1, and there is no inconformity with the test.

84: C after
The Chinese symbol has been revised as suggested.

87: an insert size
“Insersion size” has been revised as “an insert size”.

94: This sentence was somewhat confusing. I recommend rewriting it so it is clearer, e.g. : "25x coverage of the longest corrected reads was extracted with Perl scripts and assembled"
This sentence has been revised as “25x coverage of the longest corrected reads was extracted with Perl scripts and assembled”.

110: All 15 RNA-seq libraries were mapped to the assembly
This sentence has been revised as “All 15 RNA-seq libraries were mapped to the assembly”.

115: low quality variants
“Variations” has been revised as “variants”.
116: unique
“Uniq” has been revised as “unique”.

117: "error rate was calculated as the ration of double variation (1/1 and 1/2) number" - This is very confusing and needs to be rewritten.
This sentence has been revised as “error rate was calculated as the average number of single-nucleotide polymorphisms (SNP) and indels that appear at both alleles (labeled as 1/1 and 1/2 in Table 5) per

base”.

127: "the S. grosvenorii genome sequences were subjected to 3 gene" - the S. grosvenorii genome assembly was annotated using 3
This sentence has been revised as “the S. grosvenorii genome was annotated using 3 gene prediction pipelines”.

133: "with a repeat masked genome, while repeat masking was done by RepeatMasker." - with the repeat masked genome.
This sentence has been revised as “whith the repeat masked genome”.

134: "from Hisat2 to transcriptome with the assembly as reference," - from HISAT2 using the assembly as the reference - correct other instances of Hisat2 to HISAT2
This sentence has been revised as “from HISAT2 using the assembly as the reference”, and all “Hisat2” have been corrected.

140 (and others): "non-redundant database" : be more specific such as NCBI non-redundant protein database (nr)
“Non-redundant database” has been revised as “NCBI non-redundant protein database (nr)”.

Reviewer #2 (Major momments):
1. The English must be improved, especially singular/plural verbs such as in this sentence on line 112: " ...the alignment files WAS manipulated...". I suggest that the authors ask a native English speaker to proof-read the paper.
Yes. This sentence has been revised as “the alignment files were manipulated” and we have sent the revised manuscript to English native speakers for language editing.

2. I have a few concerns about the experimental design and methods. First, quality of the assembled consensus was evaluated by mapping Illumina RNAseq reads to the consensus. Naturally only reads containing few differences would map, yielding a biased consensus quality measurement. The real consensus quality is likely lower than the authors estimated. Instead I suggest estimating the consensus quality of the assembly by mapping the assembly to the contigs from the previous Ilumina-only based assembly and evaluating the fidelity of long (10Kb+) mutual best matches.
We were not able to compare the assembly to the Illumina-only assembly, because we cannot obtain the assembly. We sent e-mails to the corresponding author and also PNAS editorial for the assembly but the authors did not provide it.
The evaluation by mapping RNA-Seq reads to the consensus was biased indeed, so we carried out the genome quality assessment by mapping our resequencing short reads and whole genome short reads released earlier to the assembly instead. The coverages of resequencing datasets were 92.99% and 90.79% of the genome assembly, so we believe that this evaluation was able to estimate the accuracy of our assembly.

3. I would like also to see how BUSCO results improved compared to initial Illumina-only assembly.
We analyzed the genome completeness after genome polishing described above, and the missing BUSCOs declined to 8.1%.
We were not able to compare the assembly to the original assembly by Itkin et al., because we cannot obtain the assembly. We sent e-mails to the corresponding author and also PNAS editorial for the

assembly but the authors did not provide it.
In order to compare our assembly with the original assembly by Itkin et al, we aligned both our resequencing short reads and their released whole genome short reads to our assembly using BWA mem program, and estimated the average base error rates. They were all less than 1E-3 when using the two datasets as Table 5 showed in the manuscript, which suggested a high-quality assembly. The differences of base error rates between our resequencing data and the one released earlier were probably due to the variety difference.

Reviewer #2 (Minor comments):

Authors do not have to satisfy these comments for publication -- these are merely suggestions. One other reason I am concerned about the consensus quality is that the genome is not inbred, and 73x total PacBio coverage (which works out to about 37x per haplotype) may not be enough to generate high enough consensus quality in regions of high heterozygosity from PacBio -only data. I would recommend getting some 60-100x whole genome Illumina data for the same sample and polishing the assembly with Pilon.
We thank the reviewer for this suggestion, and we have gotten 50G (over 100x) whole genome Illumina short reads for variety Qingpiguo and used this dataset to polish the assembly, and the genome quality has been improved to a certain extent.

Also for the same reason using only 25x of the corrected reads may not be optimal -- I suspect assembly contiguity could be better it 35 or 40x of the longest corrected reads are used.
As a matter of fact, we tried some different scales of corrected long reads to assemble the genome, while 25x was the best dataset as the result assembly had the longer total size and contig N50 length.
Corrected_40X_long_reads Corrected_25X_long_reads
Number_of_contigs 4,282 4,128
Total_size(bp) 465,219,980 467,072,951
Contig_N50(bp) 349,315 433,684
Longest_contig(bp) 7,653,141 7,657,852
GC_content 33.60% 33.57%

Close
