# Supplementary material for: Improved de novo genome assembly and analysis of the Chinese cucurbit Siraitia grosvenorii, also known as monk fruit or luo-han-guo
Source: Gigascience. 2018 Jun 8;7(6):giy067. doi: 10.1093/gigascience/giy067 (PMC6007378; doi:10.1093/gigascience/giy067)
Supplement: Response_to_Reviewer_Comments_Revision_1.pdf [file giy067_response_to_reviewer_comments_revision_1.pdf]

Author's Response To Reviewer Comments

Close

Hans Zauner
Assistant Editor
GigaScience

Dear Dr. Zauner,

Thank you for handing out our revised manuscript entitled “Improved de novo genome assembly and analysis of the Chinese cucurbit Siraitia grosvenorii, also known as monk fruit or luo-han-guo” (GIGA-D-17-00311). We have removed the highlights of changes as your request and updated the files the data curator suggested in the e-mail at the first time he contacted us. We also added the citation of GigaDB in this revised manuscript, but we still do not know the doi link. In addition, we thank the reviewer 2 for the comment of assembling long contigs using paired-end reads to measure the consensus quality of our final assembly. We did not use this method to assess the genome quality because we have measured the sequence accuracy using paired-end reads mapping to the assembly, and the second method was widely used in genome quality assessment.

Close
