# Supplementary material for: Improved de novo genome assembly and analysis of the Chinese cucurbit Siraitia grosvenorii, also known as monk fruit or luo-han-guo
Source: Gigascience. 2018 Jun 8;7(6):giy067. doi: 10.1093/gigascience/giy067 (PMC6007378; doi:10.1093/gigascience/giy067)
Supplement: Reviewer_1_Report_(Original_Submission) -- John Hamilton — 1/8/2018 Reviewed [file giy067_reviewer_1_report_(original_submission).pdf]

Reviewer Report

Title: **Improved de novo genome assembly and analysis of the Chinese cucurbit Siraitia grosvenorii, also known as monk fruit or luo-han-guo**

Version: **Original Submission** **Date:** 1/8/2018

Reviewer name: **John Hamilton**

Reviewer Comments to Author:

The manuscript describes the Long-read sequencing, assembly, and annotation of an improved genome of the Siraitia grosvenorii, a cucurbit with medicinal properties that also produces mogrosides, a class of sweet-tasting compounds with interest in use as an artificial sweetener. A genome assembly for S. grosvenorii has been previously published in 2016 by Itkin et al. using a hybrid assembly of Moleculo Synthetic Long Reads and Illumina WGS. Itkin et al. state that the aim of generating a draft assembly was to obtain the sequence of genes in gene families of interest. Indeed, the draft genome assembly does not seem to be publicly available to download in GenBank or a data repository. The improved (and publicly available) genome assembly, annotation and analyses described in this data note will be a useful resource to the community.Comments:98: "This genome size was slightly larger than the estimated 420 Mb [8], which was probably due to the high genome heterozygosity." - A k-mer analysis or SNP density analysis should be done and included in the manuscript to substantiate this assertion. 99: Was the genome assembly polished after assembly to correct sequencing errors? This is normally done for PacBio assemblies and should be included in the methods if it was done. 105/Table 3: 13.9% missing BUSCOs seems high for a high coverage PacBio assembly. How does this compare to the original assembly by Itkin et al.? Minor issues/typos:20: platforms63: is a useful resourceTable 1: fix units in the table, they are correct in the text84: C after87: an insert size94: This sentence was somewhat confusing. I recommend rewriting it so it is clearer, e.g. : "25x coverage of the longest corrected reads was extracted with Perl scripts and assembled"110: All 15 RNA-seq libraries were mapped to the assembly 115: low quality variants116: unique117: "error rate was calculated as the ration of double variation (1/1 and 1/2) number" - This is very confusing and needs to be rewritten. 127: "the S. grosvenorii genome sequences were subjected to 3 gene" - the S. grosvenorii genome assembly was annotated using 3133: "with a repeat masked genome, while repeat masking was done by RepeatMasker." - with the repeat masked genome.134: "from Hisat2 to transcriptome with the assembly as reference," - from HISAT2 using the assembly as the reference - correct other instances of Hisat2 to HISAT2140 (and others): "non-redundant database" : be more specific such as NCBI non-redundant protein database (nr)

Level of Interest

Please indicate how interesting you found the manuscript: An article whose findings are important to those with closely related research interests

Quality of Written English

Please indicate the quality of language in the manuscript: Needs some language corrections before being published

Declaration of Competing Interests

Please complete a declaration of competing interests, considering the following questions:

- Have you in the past five years received reimbursements, fees, funding, or salary from an organisation that may in any way gain or lose financially from the publication of this manuscript, either now or in the future?
- Do you hold any stocks or shares in an organisation that may in any way gain or lose financially from the publication of this manuscript, either now or in the future?
- Do you hold or are you currently applying for any patents relating to the content of the manuscript?
- Have you received reimbursements, fees, funding, or salary from an organization that holds or has applied for patents relating to the content of the manuscript?
- Do you have any other financial competing interests?
- Do you have any non-financial competing interests in relation to this paper?

If you can answer no to all of the above, write 'I declare that I have no competing interests' below. If your reply is yes to any, please give details below.

I declare that I have no competing interests

I agree to the open peer review policy of the journal. I understand that my name will be included on my report to the authors and, if the manuscript is accepted for publication, my named report including any attachments I upload will be posted on the website along with the authors' responses. I agree for my report to be made available under an Open Access Creative Commons CC-BY license (http://creativecommons.org/licenses/by/4.0/). I understand that any comments which I do not wish to be included in my named report can be included as confidential comments to the editors, which will not be published.

I agree to the open peer review policy of the journal

To further support our reviewers, we have joined with Publons, where you can gain additional credit to further highlight your hard work (see: https://publons.com/journal/530/gigascience). On publication of this paper, your review will be automatically added to Publons, you can then choose whether or not to claim your Publons credit. I understand this statement.

Yes
