# Supplementary material for: Improved de novo genome assembly and analysis of the Chinese cucurbit Siraitia grosvenorii, also known as monk fruit or luo-han-guo
Source: Gigascience. 2018 Jun 8;7(6):giy067. doi: 10.1093/gigascience/giy067 (PMC6007378; doi:10.1093/gigascience/giy067)
Supplement: Reviewer_2_Report_(Original_Submission) -- Aleksey Zimin — 1/9/2018 Reviewed [file giy067_reviewer_2_report_(original_submission).pdf]

Reviewer Report

Title: Improved de novo genome assembly and analysis of the Chinese cucurbit Siraitia grosvenorii, also known as monk fruit or luo-han-guo

Version: Original Submission **Date:** 1/9/2018

Reviewer name: Aleksey Zimin

Reviewer Comments to Author:

This is a nicely written data note about an important genome. MAJOR COMMENTSI would like these comments addressed before the paper is accepted for publication1. The English must be improved, especially singular/plural verbs such as in this sentence on line 112: " ...the alignment files WAS manipulated...". I suggest that the authors ask a native English speaker to proof-read the paper. 2. I have a few concerns about the experimental design and methods. First, quality of the assembled consensus was evaluated by mapping Illumina RNAseq reads to the consensus. Naturally only reads containing few differences would map, yielding a biased consensus quality measurement. The real consensus quality is likely lower than the authors estimated. Instead I suggest estimating the consensus quality of the assembly by mapping the assembly to the contigs from the previous Ilumina-only based assembly and evaluating the fidelity of long (10Kb+) mutual best matches. 3. I would like also to see how BUSCO results improved compared to initial Illumina-only assembly. MINOR COMMENTS/SUGGESTIONSAuthors do not have to satisfy these comments for publication -- these are merely suggestions One other reason I am concerned about the consensus quality is that the genome is not inbred, and 73x total PacBio coverage (which works out to about 37x per haplotype) may not be enough to generate high enough consensus quality in regions of high heterozygosity from PacBio -only data. I would recommend getting some 60-100x whole genome Illumina data for the same sample and polishing the assembly with Pilon. Also for the same reason using only 25x of the corrected reads may not be optimal -- I suspect assembly contiguity could be better it 35 or 40x of the longest corrected reads are used.

Level of Interest

Please indicate how interesting you found the manuscript: An article whose findings are important to those with closely related research interests

Quality of Written English

Please indicate the quality of language in the manuscript: Needs some language corrections before being published

Declaration of Competing Interests

Please complete a declaration of competing interests, considering the following questions:

- Have you in the past five years received reimbursements, fees, funding, or salary from an organisation that may in any way gain or lose financially from the publication of this manuscript, either now or in the future?

- Do you hold any stocks or shares in an organisation that may in any way gain or lose financially from the publication of this manuscript, either now or in the future?
- Do you hold or are you currently applying for any patents relating to the content of the manuscript?
- Have you received reimbursements, fees, funding, or salary from an organization that holds or has applied for patents relating to the content of the manuscript?
- Do you have any other financial competing interests?
- Do you have any non-financial competing interests in relation to this paper?

If you can answer no to all of the above, write 'I declare that I have no competing interests' below. If your reply is yes to any, please give details below.

I declare that I have no competing interests

I agree to the open peer review policy of the journal. I understand that my name will be included on my report to the authors and, if the manuscript is accepted for publication, my named report including any attachments I upload will be posted on the website along with the authors' responses. I agree for my report to be made available under an Open Access Creative Commons CC-BY license (http://creativecommons.org/licenses/by/4.0/). I understand that any comments which I do not wish to be included in my named report can be included as confidential comments to the editors, which will not be published.

I agree to the open peer review policy of the journal

To further support our reviewers, we have joined with Publons, where you can gain additional credit to further highlight your hard work (see: https://publons.com/journal/530/gigascience). On publication of this paper, your review will be automatically added to Publons, you can then choose whether or not to claim your Publons credit. I understand this statement.

Yes
